# A New General Correlation for the Influence Parameter in Density Gradient Theory and Peng–Robinson Equation of State for *n*-Alkanes

**DOI:** 10.3390/molecules29235643

**Published:** 2024-11-28

**Authors:** Isidro Cachadiña, Ariel Hernández, Ángel Mulero

**Affiliations:** 1 Departamento de Física Aplicada, Universidad de Extremadura, 06006 Badajoz, Spain; mulero@unex.es; 2 Departamento de Ingeniería Industrial, Facultad de Ingeniería, Universidad Católica de la Santísima Concepción, Alonso de Ribera 2850, Concepción 4090541, Chile; ahernandez@ucsc.cl

**Keywords:** surface tension, Peng–Robinson equation of state, Density Gradient Theory, influence parameter, *n*-alkanes

## Abstract

The Density Gradient Theory (DGT) permits obtaining the surface tension by using an equation of state and the so-called influence parameter. Different correlations of the influence parameter versus temperature have been proposed, with the two-coefficient ones from Zuo and Stenby (full temperature range) and Miqueu et al. (valid for the lower temperature range) being widely used. Recently, Cachadiña et al. applied the DGT with the Peng-Robinson Equation of State to esters. They proposed a new two-coefficient correlation that uses a universal exponent related to the critical exponent associated with the dependence of coexistence densities on temperature near the critical point. When applied to *n*-alkanes, it is shown that the Cachadiña et al. correlation must be modified to improve the lower temperature range behavior. The proposed modification results in a three-coefficient correlation that includes the triple point temperature as an input parameter and incorporates the Zuo and Stenby and Miqueu et al. correlations as particular cases. Firstly, the correlation coefficients for each of the 32 *n*-alkanes considered are obtained by fitting the selected values for the surface tension obtained from different databases, books, and papers. The results obtained are comparable to other specific correlations reported in the literature. The overall mean absolute percentage deviation (OMAPD) between the selected and calculated data is just 0.79%. Secondly, a general correlation with three adjustable coefficients valid for all the *n*-alkanes is considered. Despite the OMAPD of 4.38% obtained, this correlation is discarded due to the high deviations found for methane. Finally, it is found that a new six-coefficient general correlation, including the radius of gyration as an input fluid parameter, leads to an OMAPD of 1.78% for the fluid set considered. The use of other fluid properties as an alternative to the radius of gyration is briefly discussed.

## 1. Introduction

Different processes, such as atomization, bubble and droplet formation, capillarity, detergency, formation of aerosols and sprays, injection of fuels, wetting, and some others [1,2,3,4,5,6,7] are driven by surface tension as it is an essential property of liquids. In particular, pure liquid *n*-alkanes and their mixtures are used in some industrial processes such as the use of liquefied natural gas as a fuel or the injection and atomization of fuels in engines [8,9,10,11,12,13,14,15,16,17,18,19,20,21,22,23,24].

In the petroleum industry, the surface tension of *n*-alkanes has to be considered in the enhanced oil recovery technique, where gases, as ${CO}_{2}$, of surfactants, are added to reduce the interfacial properties between crude oil and the geological reservoir [25]. Moreover, the determination of vapor–liquid equilibrium properties and the surface tension of *n*-alkanes are required in studying how to remove hydrocarbons from liquid effluents [26,27], as well as in carbon dioxide capture and storage technologies [24,28,29,30], and when used as additives in fuels [13,14,20,31,32]. In many of these applications, high temperatures and pressures are requested, but they are not always accessible. Then, accurate models or prediction methods with reasonable extrapolation and prediction capabilities are needed [24,29].

Some approaches, such as computer simulations, semi-theoretical methods, artificial intelligence, group contribution, quantitative structure–property relationships, corresponding states’ principle methods, or combinations of them, have been applied to obtain the surface tension of different kinds of liquids [33,34,35,36,37,38,39,40,41,42,43,44,45,46,47,48,49,50,51,52,53,54,55,56]. In the case of computer simulation, results available presently only lead to reproducing qualitatively the experimental values [21,29,57,58,59,60]. In the application of other approaches mentioned above, the results obtained are not entirely satisfactory due to the limited number and/or data considered and/or the fact that no selection or previous comparison between different data sources is made. This fact encouraged us to select the most appropriate ones for *n*-alkanes in a recent paper [61] and we built a complete ensemble of selected values.

The application of the Density Gradient Theory together with an analytical equation of state (DGT + EoS) is one of the most successful methods used to model surface tension and other interfacial properties [14,20,27,29,33,58,59,62,63,64,65,66,67,68,69,70]. The method connects the liquid and vapor properties of the fluid, obtained from the selected EoS, with the surface tension through a parameter called the “influence parameter”. As the value of this parameter depends on the fluid and temperature considered, it is useful to have correlations allowing its calculation from the values of some fixed properties of the fluid, i.e., its triple point temperature, acentric factor, molar volume, critical temperature, etc. [62,70,71,72]. Also, it would be desirable to have a general correlation, including general coefficients valid for at least one family of fluids, that gave accurate results and that had the predictive capacity to apply it to other fluids of the same or similar family and also to mixtures of them.

In a previous paper, Cachadiña et al. [72] studied the correlations for the influence parameter available in the literature and developed a new correlation for the influence parameter of a group of esters. The proposed analytical expression included two adjustable coefficients for each ester and a common exponent *n* for all esters. This exponent holds a physical significance as it is linked to the three-dimensional Ising universality class [73,74], which describes the phase transition near the critical point. In particular, Cachadiña et al. found the relation n=8β−3, with β the critical exponent associated to density, i.e., ($\left(\rho_{l}-\rho_{v}\right)\propto {\left[\left(T-T_{c}\right)/T_{c}\right]}^{\beta }$, with $T_{c}$ being the critical temperature and $\rho_{l}$ and $\rho_{v}$ the liquid and vapor saturation densities, respectively). The new correlation effectively replicates the surface tension of 39 esters, yielding an overall deviation of 1.37% [72].

Following a similar procedure, the main aim of this paper is to propose a general correlation for the influence parameter of *n*-alkanes. The Peng–Robinson equation of state [75,76] is used to obtain the vapor–liquid equilibrium densities for each temperature. Then, the DGT is applied to obtain the influence parameter values in a wide range of temperatures by taking the values for the surface tension selected from different data sources (databases, books, and papers) as referents.

Then, different correlations are considered to fit these data into an analytical expression that could reproduce them accurately for each fluid. Then, a generalized expression is used, which permits obtaining the influence parameter and then the surface tension of *n*-alkanes by using adjustable coefficients and some fixed thermodynamic properties for each fluid as input parameters.

This paper is organized as follows. First, the general expressions for the DGT and the Peng–Robinson EoS are introduced. Moreover, some advantages and disadvantages of using this method are summarized. Section 3 describes the surface tension data set that has been considered. In Section 4, some previous correlations to obtain the influence parameter as a function of the temperature are analyzed. Then, a new correlation is proposed for the case of *n*-alkanes. Results are shown in Section 5, and, finally, conclusions are summarized in Section 6.

## 2. Density Gradient Theory and Peng–Robinson Equation of State

The Density Gradient Theory, developed by Cahn and Hilliard [77] and later used in combination with an equation of state by Carey et al. [78], has been successfully applied for years to correlate and predict the surface tension of pure fluids and their mixtures [70,71,78,79,80,81,82,83].

The DGT + EoS method leads to the calculation of the surface tension σ at a given temperature *T* through the following expression [65,78]:

(1) $$\sigma =\int_{\rho_{V}^{0}}^{\rho_{L}^{0}}\sqrt{2c\left[f_{0}-\rho {\left(\frac{\partial f_{0}}{\partial \rho }\right)}_{T}^{0}+p^{0}\right]}d\rho ,$$

where ρ is the molar density, $\rho_{V}^{0}$ and $\rho_{L}^{0}$ are the saturated vapor and liquid molar densities at temperature *T*, $p^{0}$ is the saturation pressure, and *c* is the influence parameter, which can be considered to be only temperature-dependent.

The Helmholtz energy density $f_{0}$ is defined as:

(2) $$f_{0}\left(\rho ,T\right)=\rho f\left(\rho ,T\right),$$

where f(ρ,T) is the molar Helmholtz energy of the system. As explained in Ref. [72], the molar Helmholtz energy is the sum of the molar ideal part ($f^{id}$) and the molar residual part ($f^{r})$, that is $f=f^{id}+f^{r}$. The molar ideal part $f^{id}$ is obtained from the ideal isobaric caloric capacity $c_{p}^{id}\left(T\right)$, while the molar residual part can be determined from the analytical expression of pressure (*p*) as:

(3) $$f^{r}\left(\rho ,T\right)=\int \frac{p}{\rho^{2}}d\rho -RT\log\rho .$$

As it is demonstrated in Ref. [72], the result for the integration included in (1) is independent of the molar ideal contribution ($f^{id}$), and then, when calculating the influence parameter, it can be taken that $f_{0}\equiv f_{0}^{r}=\rho f^{r}\left(\rho ,T\right)$.

It is important to note that the DGT + EoS method presents some advantages when compared with others:

(i) It is theoretically based on the van der Waals assumption that a smooth change between liquid and vapor phases exists [77,84].
(ii) The fluid properties of the saturated single phases (vapor and liquid) are obtained from an EoS, so no other data sources or analytical expressions are necessary.
(iii) The theory allows the calculation of the surface or interfacial tension, density profiles and their thickness, the surface enthalpy and entropy, and some other properties for the adsorption processes [80].

Nevertheless, according to Liang et al., the DGT + EoS method has certain limitations [85], as it is not entirely general and does not always permit making predictions. For a given fluid, the influence parameter values or the correlations proposed are not always transferable to other fluids or temperature ranges [65,86]. Also, the required calculations are not always straightforward and must be made carefully to avoid wrong results.

Additionally, when applying the DGT + EoS, it has to be noted that [72]:

(i) Different EoS will lead to different influence parameters, even for the same fluid and temperature; (ii) the influence parameter, at a given temperature, is determined by the EoS behavior between the vapor and liquid saturated densities: this region is only considered in the Maxwell equal-area equilibrium condition; and (iii) as the argument of the square root in Equation (1) must be positive, the EoS with multiple van der Waals loops could not yield acceptable results.

Although the limitations of the DGT + EoS method are well known, its advantages surpass these, and it has been successfully applied to both pure fluids and mixtures with different EoS and influence parameter correlations [62,65,69,70,71,78,79,85,86,87,88,89,90,91,92,93,94,95].

The first application of the DGT + EoS method was made in 1978 by Carey et al. [78] using the Peng–Robinson (PR) EoS. According to Garrido et al. [65], more than 150 scientific papers related to the DGT have been published up to 2016, with the Peng–Robinson and variations of the Statistical Associating Fluid Theory (SAFT) as the most used EoS. Other authors, such as Chow et al. [87] and Larsen et al. [89], report the use of the Soave–Redlich–Kwong method and Cubic-Plus-Association as other widely used alternatives.

The EoS chosen in this work to model the bulk properties of the considered *n*-alkanes is the Peng–Robinson one [76], known as PR78, as it yields more accurate vapor pressure predictions for heavy hydrocarbons [33,96,97,98].

The PR78 EoS is written as:

(4) $$p=\frac{RT}{v-b}-\frac{a}{{\left(v+b\right)}^{2}-2b^{2}},$$

where $v=\rho^{-1}$ is the molar volume, *T* the absolute temperature, *R* the ideal gas constant. The parameters *a* and *b* (known as cohesive and covolume, respectively) are related to the critical temperature $T_{c}$, critical pressure $p_{c}$, and acentric factor ω, as [76,98]:

(5) $$a=0.45724\frac{{\left(RT_{c}\right)}^{2}}{p_{c}}\alpha \left(T\right)$$

(6) $$b=0.07780\frac{RT_{c}}{p_{c}}$$

(7) $$\alpha \left(T\right)={\left[1+m\left(1-\sqrt{\frac{T}{T_{c}}}\right)\right]}^{2}$$

(8) $$m=\left\{\begin{array}{cc} 0.37464+1.5422\omega -0.26992\omega^{2} & \;\mathrm{if}\;\omega \leq 0.491\\ 0.379642+1.48503\omega -0.164423\omega^{2}+0.016666\omega^{3} & \;\mathrm{if}\;\omega >0.491 \end{array}\right.$$

with α(T) being known as the thermal cohesion function.

The main improvement in the PR78 EoS with respect to the original one [75] is the use of different correlations for *m* according to smaller or larger values of the acentric factor. As it is known, for the heaviest *n*-alkanes, the acentric factors are larger. So, Equation (8) permits us to obtain better results for the properties of the *n*-alkanes with a higher number of carbons when compared with other previous EoSs [76,96,98].

Once the EoS has been selected, the next step in applying the DGT + EoS method is to consider a set of data for the surface tension of the fluids considered. The procedure followed and the final data selection are explained in the next Section.

## 3. Sources of Data

Surface tension data can be found in databases, books, and papers. Still, before use, they have to be screened and selected, as sometimes there is apparent disagreement between data from different sources [61,99,100,101,102,103].

Recently, Mulero et al. [61] performed an extensive search to collect the surface tension data available for 33 *n*-alkanes using the DIPPR (year 2020) [104] and DETHERM [105] databases and Wohlfarth and Wohlfarth’s books [106,107,108] as primary sources and adding some very recently published data. After a screening process, they finally built a database containing 2561 values. Then, specific correlations (containing two, four, or six adjustable coefficients for each fluid) were proposed to correlate the surface tension data with temperature. These correlations reproduce the selected data with mean absolute percentage deviations for each fluid below 2.1% and percentage deviations below 10% except for nine data points (which are close to the critical point, where percentage deviations are high no matter the model or correlation used) [61]. Also, from this work, it is clear that the primary source of the deviations is the data dispersion rather than a bad functional form, so it is not expected to find a specific or general correlation yielding lower mean absolute deviations than the ones reported by Mulero et al. [61].

This work considers an upgraded version of the DIPPR (year 2022) [104] database. The main changes from the version used by Mulero et al. [61] are minor corrections of the critical, triple, and normal boiling point temperatures and the addition of some predicted surface tension data obtained by using Sugden’s correlation [109]. Thus, finally, 2681 values of surface tension data have been considered in this work.

**Figure 1:**
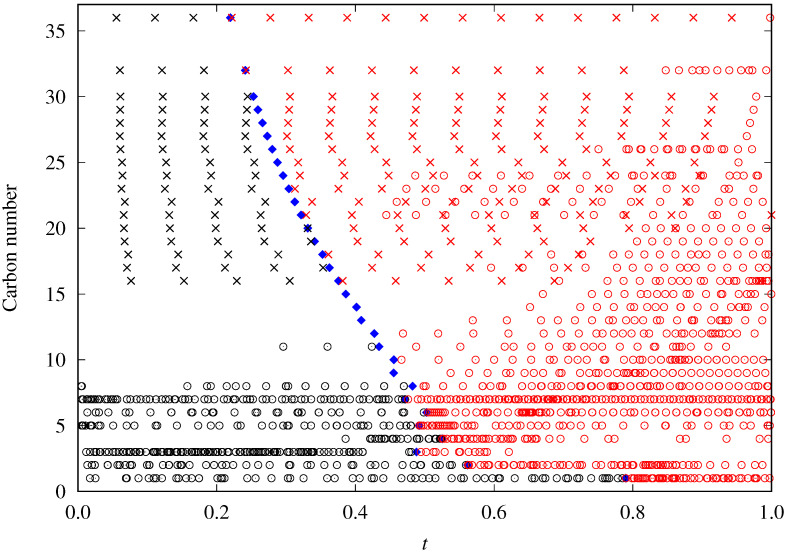
Surface tension data availability as a function of the reduced temperature *t* and carbon number of the studied *n*-alkanes. Red symbols correspond to the data below the normal point temperature. Crosses (×) are those data predicted using Sugden’s correlation, whereas circles (∘) represent the rest of the selected data. Blue diamonds correspond to each fluid’s reduced normal boiling point temperature ($t_{b}$).

In Figure 1, the reduced temperature location of the available data as a function of the carbon number is shown. It can be seen that, only for the lower *n*-alkanes, there are surface tension values above the normal boiling point different from Sugden’s DIPPR data. Then, the results obtained for these heavier *n*-alkanes must be taken cautiously.

## 4. New Correlation for the Reduced Influence Parameter

To obtain a correlation for the influence parameter in *n*-alkanes, the following step is to calculate it at each temperature, taking as a referent the selected values for the surface tension. Thus, from Equation (1), the value of the influence parameter, $c(T_{i})$, can be obtained at each temperature at which a surface tension datum ($\sigma_{i}$) is available, such as [72]:

(9) $$c\left(T_{i}\right)=\frac{\sigma_{i}^{2}}{{\left[\int_{\rho_{V}^{0}}^{\rho_{L}^{0}}\sqrt{2\left[f_{0}-\rho {\left(\frac{\partial f_{0}}{\partial \rho }\right)}_{T}^{0}+p^{0}\right]}d\rho \right]}^{2}}.$$

To take into account the parameters of the selected EoS and to facilitate the calculations, a reduced influence parameter ($c^{*}$) is defined as [70,71,79,110]:

(10) $$c^{*}=\frac{c}{ab^{2/3}},$$

with *a* and *b* being the parameters of the EoS (Equations (5) and (6), respectively) and $c^{*}$ being calculated in mol^2/3^.

For convenience, and following the same procedure as in a previous paper [72], we have used the following dimensionless reduced temperature (*t*):

(11) $$t=\frac{T_{c}-T}{T_{c}-T_{t}}.$$

Thus, *t* takes values from 0 (critical point) to 1 (triple point temperature). In the same way, the reduced boiling point temperature can be defined as:

(12) $$t_{b}=\frac{T_{c}-T_{b}}{T_{c}-T_{t}},$$

with $T_{b}$ being the normal boiling point temperature.

**Figure 2:**
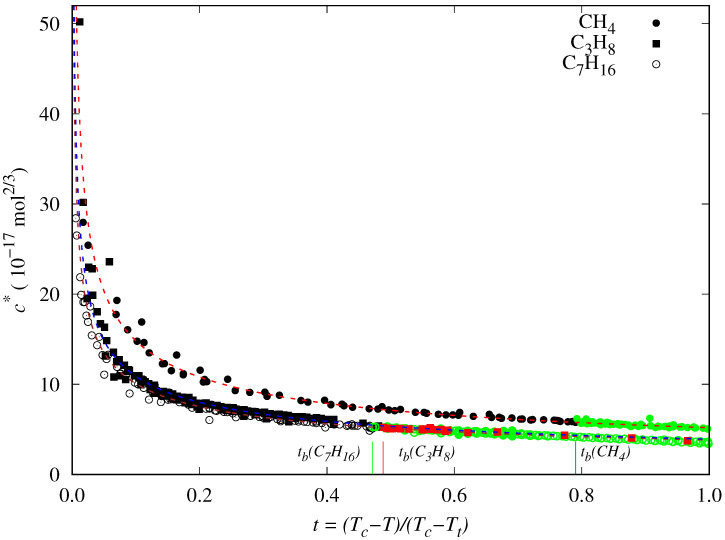
Dependency of the reduced influence parameter ($c^{*}$) with respect to reduced temperature (*t*) for methane, *n*-propane, and *n*-heptane. The data at temperatures between the triple and normal boiling points (tb≤t≤1) are colored (green for methane and heptane, and red for propane). Lines are the fit of the reduced influence parameter of each fluid to the Zuo and Stenby correlation given by Equation (14).

Some examples of the behavior of the reduced influence parameter versus the reduced temperature are shown in Figure 2. In particular, results for methane, *n*-butane, and *n*-heptane are displayed, which were calculated using the surface tension data previously selected and screened. Reduced boiling temperatures are marked as vertical lines. The chosen data at temperatures above the boiling point are shown in black, whereas those at lower temperatures are colored.

In a previous paper, we considered the dependence of $c^{*}$ versus *t* for several esters [72], and analyzing their behavior, we proposed a new correlation. We realize some common features by comparing those previous results for esters with those observed in Figure 2 for some *n*-alkanes. In particular:

- $c^{*}$ decreases almost linearly in the range [$t_{b},1$], so it could be fitted to a linear expression:
  (13) $$c^{*}\left(t\right)=m_{2}\left(t-1\right)+m_{1},$$
  where $m_{2}<0$ is the slope and $m_{1}=c^{*}\left(1\right)>0$ is the value of the reduced influence parameter at the triple point temperature.
- Near the critical point (t≃0), $c^{*}$ tends to infinity. To reproduce this behavior, an analytical expression such as the one proposed by Zuo and Stenby [70] has to be used:
  (14) $$c^{*}\left(t\right)=At^{n},\;\mathrm{with}\;n<0,$$

By combining both behaviors, Cachadiña et al. [72] have proposed a new analytical expression to calculate accurately the surface tension of esters:

(15) $$c^{*}\left(t\right)=m_{0}\left(t^{n}-1\right)+m_{1}.$$

They have shown that the value n=−0.392 can be considered a universal exponent and that it is related to the value for the critical exponent (β=0.326) associated with the density at the vapor–liquid equilibrium curve as n=8β−3 [73,74] The results obtained in Ref. [72] show that, at least for esters, the accuracy of this expression is similar to the one from Zuo and Stenby, Equation (14). Even though they have the same number of adjustable coefficients, the new proposal is linear for $m_{0}$ and $m_{1}$, so it is more straightforward to determine them when the reduced influence parameter is fitted using a least squares minimization method.

Nevertheless, when Zuo and Stenby’s (Equation (14)) or Cachadiña et al.’s (Equation (15)) correlations are applied to fluids for which surface tension data are available in a wide low-temperature range and they are linear with the reduced temperature, as is the case of a majority of *n*-alkanes, the obtained results are not so accurate.

**Figure 3:**
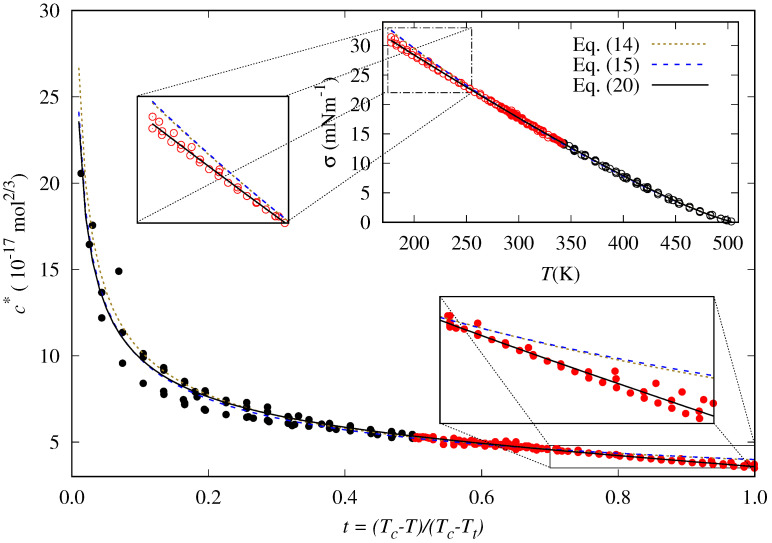
Reduced influence parameter and surface tension for *n*-hexane (points) and our fits to the Zuo and Stenby correlation [35], Equation (14) (dotted lines); Cachadiña et al. proposal [72], Equation (15) (dashed blue lines); and the new correlation proposed in this work, Equation (20) (solid lines). The data at temperatures below the normal boiling point are shown in red. Results for the lower temperatures, i.e., higher reduced temperatures, are more clearly shown in the insets.

As shown in Figure 3, in the case of *n*-hexane, for instance, the Equations (14) and (15) cannot reproduce accurately the behavior of the reduced influence parameter at low temperatures (T<250 K or t>0.7, approximately), and consequently, the surface tension values are overestimated. These two correlations cannot appropriately describe the data trend in this temperature range.

The observed behavior suggests that the second derivative of the reduced influence parameter should be near zero in the temperature range from the triple point to normal boiling point temperatures. That is:

(16) $$\frac{d^{2}c^{*}}{dt^{2}}≃0\;t_{b}>t\geq 1,$$

It is easy to show that neither the Zuo and Stenby or the Cachadiña et al. correlations fulfill this condition, as their second derivatives at t=1 are An(n−1) and $m_{0}n\left(n-1\right)$, respectively.

This work aims to propose a correction term, η(t), to the expression proposed by Cachadiña et al., Equation (15), to accurately reproduce the behavior of the reduced influence parameter at low temperatures. Thus, the new proposal is written as:

(17) $$c^{*}\left(t\right)=m_{0}\left(t^{n}-1\right)+m_{1}+\eta \left(t\right).$$

with the following conditions at t=1 (triple point temperature):

(18) $$\begin{array}{ccccc} c^{*}\left(1\right)= & m_{1} & \Rightarrow & \eta (1)= & 0,\\ \; & \; & & \; & \\ \frac{dc^{*}\left(t\right)}{dt}{|}_{t=1}= & m_{2} & \Rightarrow & \frac{d\eta (t)}{dt}{|}_{t=1}= & m_{2}-nm_{0},\\ \; & \; & & \; & \\ \frac{d^{2}c^{*}\left(t\right)}{dt^{2}}{|}_{t=1}= & 0 & \Rightarrow & \frac{d^{2}\eta \left(t\right)}{dt^{2}}{|}_{t=1}= & -m_{0}n\left(n-1\right). \end{array}$$

The simplest analytical form of η(t) is the second-order polynomial:

(19) $$\eta \left(t\right)=q_{0}+q_{1}\left(t-1\right)+q_{2}{\left(t-1\right)}^{2},$$

that leads to the following analytical expression for the reduced influence parameter:

(20) $$c^{*}\left(t\right)=m_{0}\left(t^{n}-1\right)+m_{1}+\left(m_{2}-nm_{0}\right)\left(t-1\right)-\frac{1}{2}n\left(n-1\right)m_{0}{\left(t-1\right)}^{2},$$

where the value n=−0.392 is fixed accordingly to the proposal of Cachadiña et al. [72]. As required in Equation (18), $m_{1}$ and $m_{2}$ are, respectively, the value and the slope of the reduced influence parameter at the triple point temperature (t=1).

It is essential to consider that the coefficients $m_{0}$, $m_{1}$, and $m_{2}$ must fulfill some constraints: since the influence parameter is positive, both $m_{0}$ and $m_{1}$ must be positive. On the other hand, at lower temperatures, it is observed that the reduced influence parameter follows a straight line with a negative slope [71], so $m_{2}$ has to be negative.

It is worth noting that when $m_{0}=0$, Equation (20) results in:

(21) $$c^{*}\left(t\right)=m_{1}+m_{2}\left(t-1\right),$$

which is equivalent to the linear correlation proposed by Miqueu et al. [71]:

(22) $$c^{*}=q_{1}t^{\prime }+q_{0},$$

when using PR EoS with volume translation [111]. In Equation (22), $q_{0}$ and $q_{1}$ are adjustable coefficients, and $t^{\prime }=1-T/T_{c}$ is a reduced temperature. The following relations between $q_{0}$, $q_{1}$, $m_{0}$, and $m_{1}$ are obtained easily when comparing (21) and (22):

(23) $$q_{0}=m_{1}-m_{2}.$$

(24) $$q_{1}=\frac{m_{2}}{1-\frac{T_{t}}{T_{c}}}.$$

Once the analytical expression given in Equation (20) has been proposed, the values of the coefficients must be determined. This work considers a comprehensive data set of surface tension values for 32 *n*-alkanes, and the results are shown and discussed in the next section.

## 5. Results and Discussion

**Figure 4:**
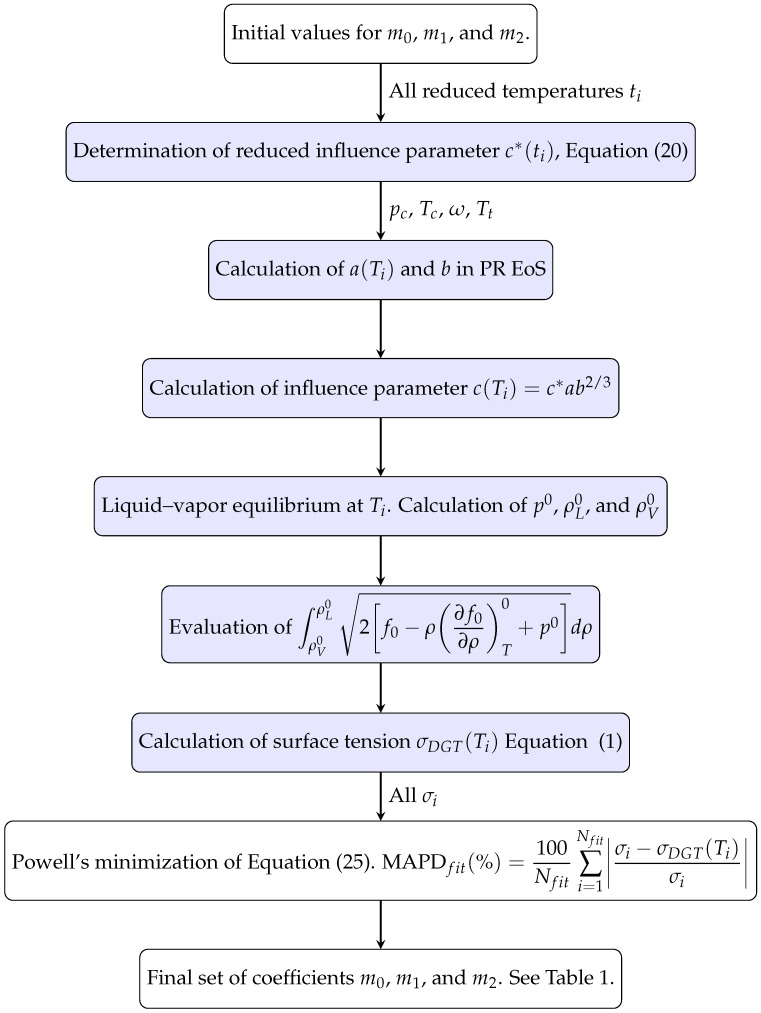
Flowchart describing how the coefficients $m_{0}$, $m_{1}$, and $m_{2}$ in Equation (20) are determined for a given fluid. The fluid parameters are $p_{c}$, $T_{c}$, ω, and $T_{t}$. The colored boxes indicate the steps for obtaining the predicted $\sigma_{DGT}$ at a temperature $T_{i}$ when $m_{0}$, $m_{1}$, and $m_{2}$ are known.

The values of coefficients $m_{0}$, $m_{1}$, and $m_{2}$ in Equation (20) have been obtained by minimizing the mean absolute percentage deviation (MAPD_*fit*_) of every fluid:

(25) $${\mathrm{MAPD}}_{fit}\left(\%\right)=\frac{100}{N_{fit}}\sum_{i=1}^{N_{fit}}\left|\frac{\sigma_{i}-\sigma_{DGT}\left(T_{i}\right)}{\sigma_{i}}\right|,$$

wher $N_{fit}$ is the number of surface tension values selected for fitting that fluid. $\sigma_{i}$ is the surface tension datum at temperature $T_{i}$, and $\sigma_{DGT}\left(T_{i}\right)$ is the calculated surface tension at the same temperature (see Figure 4 for a flowchart describing the procedure). As is well known, the surface tension must be precisely zero at the critical point, and it takes values very near zero at temperatures closer to it. As a consequence, when using percentage deviations, it has to be clear that the same absolute deviation, $\Delta \sigma =\sigma_{i}-\sigma_{DGT}\left(T_{i}\right)$, will produce a higher percentage deviation near the critical point temperature as the denominator in the ratio Δσ/σ is very close to zero near this point [72]. Thus, for instance, if σ = 0.001 mNm^−1^ and Δσ = 0.001 mNm^−1^, the percentage deviation will be 100%. This effect is quite apparent when correlating data from different authors near the critical point, where no matter the correlation used, high-percentage deviations are always observed [61,99]. For this reason, the data very close to $T_{c}$ (t∼0) are not taken into account in the fitting process, as they could bias the fitting results with a significant contribution to the sum in Equation (25). Thus, only data with t≥0.02 and those from Sugden’s correlation (t≥0.02) are considered in determining the specific correlation coefficients. The fitting set has $N_{fit}=2652$ data out of N=2680 available data. The statistical figures discussed below will be calculated for the whole and fitting data sets.

**Figure 5:**
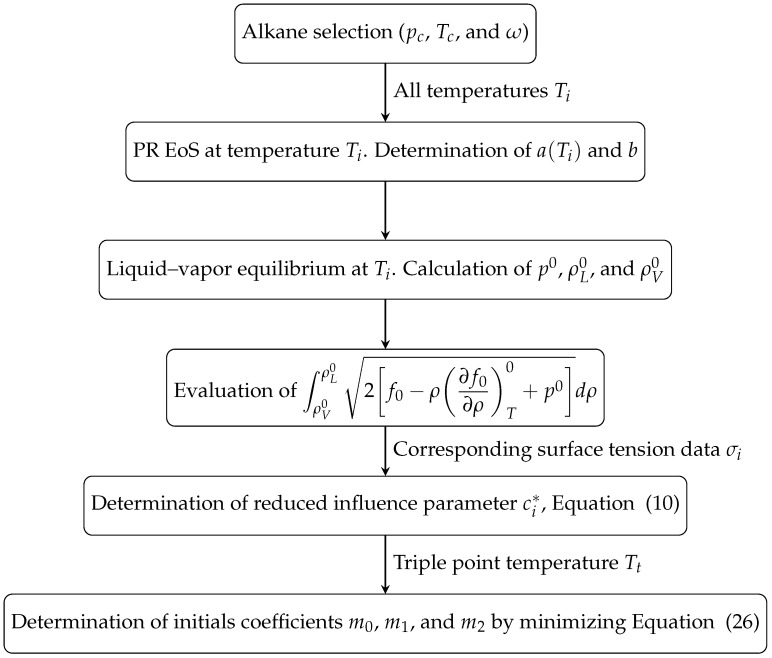
Flowchart describing the determination of the initial values for the coefficients $m_{0}$, $m_{1}$ and $m_{2}$, of the reduced influence correlation given by (20).

As the objective function Equation (25) is not linear in the adjustable coefficients, a careful choice of its initial values has to be taken. Since the expression for $c^{*}$ in Equation (20) is linear in $m_{i}$ (i=0,1,2), the initial values for $m_{i}$ have been obtained by using a linear least squares method [112] using the following merit function:

(26) $$S^{2}=\sum_{i=1}^{N_{fit}}{\left[\frac{c_{i}^{*}-c_{model}^{*}\left(t_{i}\right)}{c_{i}^{*}}\right]}^{2},$$

and the flowchart given in Figure 5, whichsummarizes the steps required.

Then, these values were used in Powell’s minimization algorithm [112] to find the minimum MAPD_*fit*_ (Equation (25)). To prevent Powell’s method from falling into a local minimum, random displacements were given to the best values for the adjustable coefficients found, and then a new minimization was carried out. After 50 random displacement iterations, the coefficients of the lowest MAPD_*fit*_ found are saved.

The statistical figures discussed in the next two sections are defined for each fluid, considering all the available data (*N*) and regardless of the number of data fitted (see Table 1). The mean absolute percentage deviation (MAPD):

(27) $$\mathrm{MAPD}\left(\%\right)=\frac{100}{N}\sum_{i=1}^{N}\left|\frac{\sigma_{i}-\sigma_{DGT}\left(T_{i}\right)}{\sigma_{i}}\right|,$$

the mean deviation (MD):

(28) $$\mathrm{MD}\left(\%\right)=\frac{100}{N}\sum_{i=1}^{N}\frac{\sigma_{DGT}\left(T_{i}\right)-\sigma_{i}}{\sigma_{i}},$$

and the maximum absolute percentage deviation (PD_m_):

(29) $${\mathrm{PD}}_{m}=100\;\cdot \;\max_{i=1,\ldots ,N}\left|\frac{\sigma_{i}-\sigma_{DGT}\left(T_{i}\right)}{\sigma_{i}}\right|.$$

In the following subsections, the values for the adjustable coefficients of the specific model for each fluid are given, and their variation is analyzed. Then, the accuracy of these proposed correlations will be discussed. Finally, the last section is devoted to proposing a new general correlation valid for all the considered *n*-alkanes and studying its results. As a first step, the possibility of having a general set of coefficients $m_{0}$, $m_{1}$, and $m_{2}$ valid for the *n*-alkanes family will be considered. Then, a general correlation, keeping as many constant $m_{i}$ coefficients as possible and yielding low deviations, will be studied as a second step. In this case, the use of some fluid properties as input parameters for the correlations of the non-constant $m_{i}$ coefficients will be considered.

### 5.1. Adjustable Coefficients for the Specific Correlation

**Figure 6:**
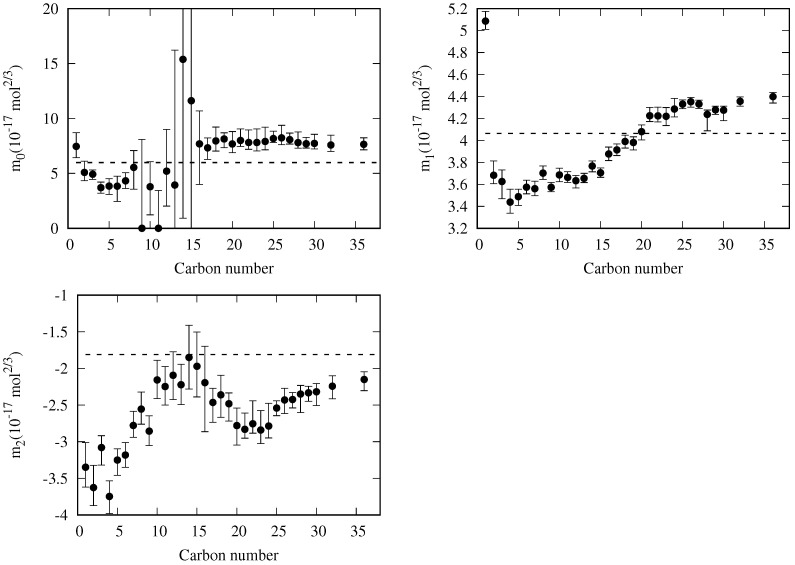
Values of the adjustable coefficients ($m_{0}$, $m_{1}$ and $m_{2}$) as a function of the carbon number of the *n*-alkanes. The vertical bars indicate the range within the resulting MAPD increases less than 0.25% from the lowest value. The dashed lines corresponds to the constant values $m_{0}=5.983$, $m_{1}=4.060$, and $m_{2}=-1.810$ (in $10^{-17}$ mol^2/3^ units) of the simplest global model considered in Section 5.3.

As a first step to study whether a global correlation for the $m_{i}$ can finally be found, it is interesting to evaluate how the variation of the value of a given $m_{i}$ coefficient influences the resulting MAPD. Thus, we have calculated the displacements $\Delta m_{i}^{+}$ and $\Delta m_{i}^{-}$ that increase the MAPD value by 0.25%, keeping the other coefficients fixed. In Figure 6, the values of $m_{0}$, $m_{1}$, and $m_{2}$ are plotted as a function of the carbon number of each *n*-alkane, with the vertical bars indicating the ranges $m_{i}-\Delta m_{i}^{-}$ and $m_{i}+\Delta m_{i}^{+}$. The numerical values of $m_{i}$ and the $\min(\Delta m_{i}^{-},\Delta m_{i}^{+})$, MAPD, maximum percentage deviation (PD_m_), and reduced temperature $t_{\mathrm{PDm}}$ where the maximum deviation is reached, are compiled in Table 1.

It is noteworthy that *n*-nonane and *n*-undecane have their $m_{0}=0$. This value results from the lack of data at temperatures above the normal boiling point (see Figure 1). Thus, when plotting the reduced influence parameter versus the reduced temperature for both fluids, the observed values follow a straight line with no appreciable curvature. Since the constraint $m_{0}\geq 0$ is set in the fitting process, the result is the analytical expression mentioned in Equation (21). As shown in Table 1, *n*-nonane and *n*-undecane $m_{0}$ can rise up to 8.1 and 3.4 × $10^{-17}\;{\mathrm{mol}}^{2/3}$, respectively, without increasing the resulting MAPD by more than 0.25%.

The error bars associated with the obtained values for the adjustable coefficients of these fluids suggest that there is some room to choose other values for the adjustable coefficients that lead to a non-zero $m_{0}$ value, which could lead to a suitable extrapolation to higher temperatures, which will be discussed in the next section.

For the other *n*-alkanes considered, the $m_{0}$ values decrease with the carbon number down to *n*-butane. The trend for the heavier fluids seems to be compatible with a plateau starting from the *n*-alkane with 16 carbons. Unfortunately, this observation is biased by the fact that the $m_{0}$ values are determined mainly by the high-temperature range, and for these fluids, all the available data in this range (see Figure 1) are the predictions made by the DIPPR project [104] using Sugden’s correlation.

The narrow bars for $m_{1}$ observed for all the *n*-alkanes in Figure 6 are related to the role that $m_{1}$ plays in the proposed correlation, as the surface tension values at lower temperatures mainly determine it. As there is a high data availability in the temperature range between the triple and normal boiling points, a slight variation of $m_{1}$ will lead to a considerable change in the predicted values for this range, and so the observed narrow bars are expected.

The highest value for $m_{1}$ is obtained for methane, decreasing to *n*-butane and increasing for higher *n*-alkanes. On the other hand, for carbon numbers higher than 25, the value of $m_{1}$ could be regarded as a constant.

Coefficient $m_{2}$ is related to the slope of the reduced influence parameter at the triple point temperature. The tendency observed in Figure 6 is almost a linear behavior up to carbon number 15. For higher carbon numbers, the observed behavior could be due to the DIPPR predictions, so we need to take them with some caution.

Finally, it is necessary to stress that the $m_{i}$ values reported in this work can be used to match the observed behavior for *n*-alkanes with a high degree of accuracy. Nevertheless, it has to be clear that, for carbon numbers greater than 15, the given correlation will reproduce mostly the DIPPR predictions. When new data become available for one of these fluids, a new fit is expected to yield a certain change in the corresponding $m_{0}$ and $m_{2}$ values.

### 5.2. Accuracy of the Proposed Specific Model

By using Equation (20) and the values for the adjustable coefficients obtained for each fluid, the MAPDs, MDs, and PD_m_ were calculated. Results are shown in Table 1 (remember that these values are obtained with all the data available for each fluid and not only with the data considered in the fit).

The resulting deviation values are of the same order as those reported by Mulero et al. for *n*-alkanes [61] when using the Guggenheim–Katayama correlation with two, four, or six fitting coefficients for each *n*-alkane. For example, making a comparison for methane, the use of the Guggenheim–Katayama correlation with four adjustable coefficients leads to an MAPD of 0.93% and PD_m_ of 9.68% [61], while using the here-proposed correlation with three adjustable coefficients, the obtained deviations are MAPD = 0.97% and PD_m_ = 8.5%.

**Figure 7:**
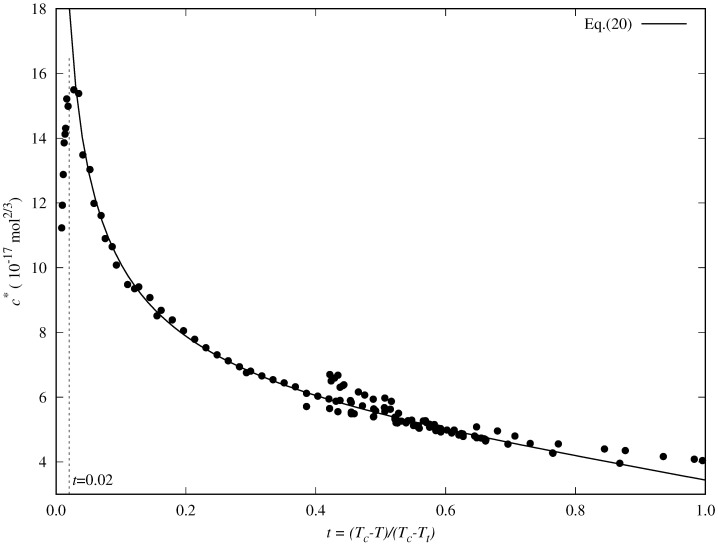
Reduced influence parameter versus the reduced temperature for *n*-butane (points) and results obtained with the proposed correlation, given in Equation (20).

The highest PD_m_ value found here, 48.9%, is obtained for *n*-butane, while Mulero et al. reported a PD_m_ value of 11.70% using six adjustable coefficients for this same fluid [61]. This highest PD_m_ value is located near the critical point temperature ($t_{{\mathrm{PD}}_{m}}=0.01$). As shown in Figure 7, this is due to the anomalous behavior of the reduced influence parameter observed for this fluid, which decreases sharply at temperatures t≤0.02. Consequently, a high disagreement between the correlation predicted data and the surface tension values is obtained in this temperature range, i.e., near the critical point. Note that for the first eight *n*-alkanes, the results for t<0.02 are extrapolated values, and the PDm of five out of the eight first *n*-alkanes are located in this range.

As the surface tension values near the critical point are subject to high uncertainty, it is interesting to check what values for MAPD and PD_m_ are obtained when a narrower temperature range is considered but keeping the $m_{i}$ values compiled in Table 1.

**Figure 8:**
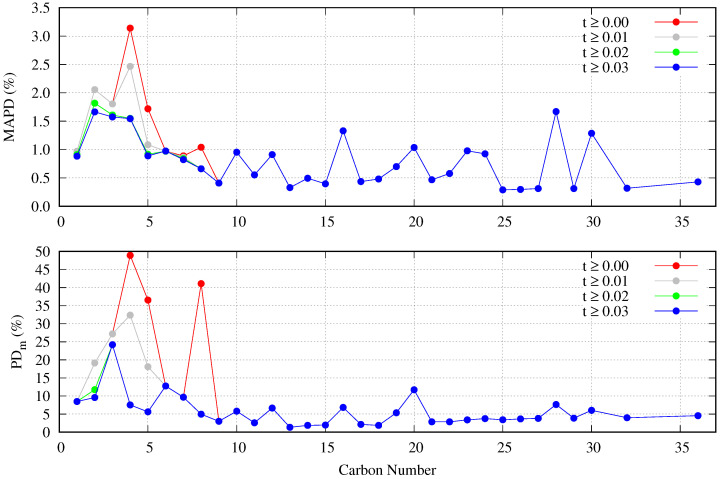
MAPD and PD_m_ as a function of carbon number when different temperature ranges are taken into consideration.

As shown in Figure 8, the maximum value of 48.9% for *n*-butane is lowered down to 7.54% when only data with t≥0.02 are considered. No significant reduction is obtained when t≥0.03, where only the PD_m_ of ethane is affected. On the other hand, the MAPDs calculated in the range t≥0.02 yield values below 2% in all cases, a reasonable value since their origin is due to the disagreement between the different sources of surface tension data rather than the analytical form of the proposed correlations.

### 5.3. General Correlation

The lack of experimental data above the normal boiling point, especially for the higher *n*-alkanes (see Figure 1), suggests the importance of the development of a general correlation applicable to all of them and that permits us to obtain predicted values. The DIPPR project, for example, includes in its database some predicted data based on the application of Sugden’s correlation [109]. This correlation establishes a relation between surface tension and the fourth power of the liquid and vapor density difference. These data should not be considered when developing a new general correlation.

Before obtaining a general correlation, we need to make some considerations:

1. The DIPPR data predicted using Sugden’s correlation will not be considered in the new global correlation, and only data in the range t≥0.02 will be considered in the coefficient determination of the global correlation. The number of available data and fitting data for each fluid are compiled in Table 2.
2. There are some fluids (see Figure 1) for which a considerable number of fitting data are available (i.e., *n*-heptane and *n*-hexane with 357 and 269 data, respectively), whereas in other cases the number of data is one (*n*-hexatriacontane and others). To have a suitable general correlation not biased by the data availability, the adjustable coefficients will be obtained by minimizing the overall mean absolute percentage deviation (OMAPD_*fit*_), defined as:
  (30) $${\mathrm{OMAPD}}_{fit}\left(\%\right)=\frac{1}{N_{fluids}}\sum_{k=1}^{N_{fluids}}{\mathrm{MAPD}}_{fit,k},$$
  where MAPD_*fit*,*k*_ is the mean absolute percentage deviation of the fluid *k*, defined in Equation (25), but taking $N_{fit}$ from Table 2, and with $N_{fluids}$ the number of fluids (32 in these cases). Thus, the coefficients of the general correlation will be determined with $N_{fit}=2427$ data, with a weighing scheme depending on the fitting data of each fluid.

As a first approximation, a general correlation where all the $m_{i}$ coefficients are regarded as constant is explored. The coefficient values that minimized the OMAPD_*fit*_, defined in Equation (30), are: $m_{0}=5.983$, $m_{1}=4.060$, and $m_{2}=-1.810$ (all of them in $10^{-17}$ mol^2/3^ units). These values, which are shown as dashed lines in Figure 6, yield the OMAPDs of the whole and fitting data sets of 4.38% and 3.35%, respectively (see Table 2). When considering the overall mean deviation for the whole and fitting sets, defined as:

(31) $${\mathrm{OMD}}_{\mathrm{whole}/fiting}\left(\%\right)=\frac{1}{N_{fluid}}\sum_{k=1}^{N_{fluid}}{\mathrm{MD}}_{k,\mathrm{whole}/fitting}$$

the values obtained OMD_whole_ =−1.38% and OMD_*fit*_ =−0.53%, respectively. This shows that the surface tension is under-predicted in most fluids (i.e., MD is negative in most cases).

**Figure 9:**
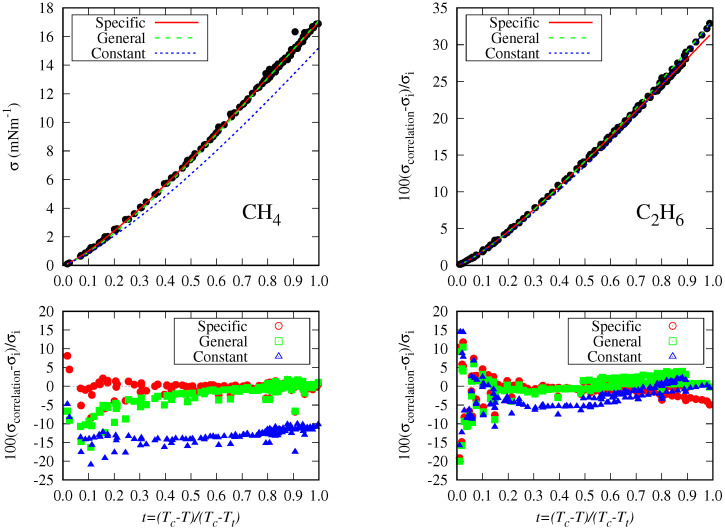
Surface tension data for methane and ethane (circles) and the specific, general, and constant correlations (lines) versus the reduced temperature. The percentage deviations for each correlation are represented below the surface tension figures with different colors and symbols.

As expected, this most straightforward correlation gives poor results when compared to the specific correlations given in Table 1, but these results are not so bad when considering the difference in the number fitting coefficients: 96 vs. 3. Indeed, the MAPD_*fit*_ values are less than 5.0% with the only exception of methane (see Figure 9), for which the largest MAPD and MD deviations are found. In other fluids, i.e., ethane (see Figure 9), the constant correlation yields reasonable results despite the low number of fitting coefficients used.

It is crucial to take into account that, in some cases, the deviations are not only due to the behavior of the model but also to the disagreement between the data for the surface tension obtained by different authors for the same fluid and temperature range (the previous figures show several examples). As expected, despite the absolute deviations being low at high temperatures (*t* near zero), the percentage deviations are high, as is the case for any other models or correlations [61].

In addition, it must be pointed out that a new coefficient fit, excluding methane, could lead to lower MAPDs for the other *n*-alkanes. In this way, two 3-coefficient sets (3 for methane and 3 for the other *n*-alkanes) could be proposed, but, as is shown below, it is possible to find a 6-coefficient general correlation depending on some fluid parameter and giving low MAPDs with good extrapolation features.

One of the present work aims is to give a simple general correlation for the surface tension of *n*-alkanes that could correlate the available surface tension data with high accuracy and having good extrapolation capability. That kind of correlation is usually written as a function of some fluid properties with fixed values, such as the critical pressure, acentric factor, or others [113]. In this work, we have considered all the properties in Table 3, whose values were obtained from the DIPPR [104] database. Nevertheless, it is worth noting that some of the fluid properties in the DIPPR database are also predicted, and it is especially true for the higher *n*-alkanes, so the predictions made for these fluids have to be taken cautiously.

**Figure 10:**
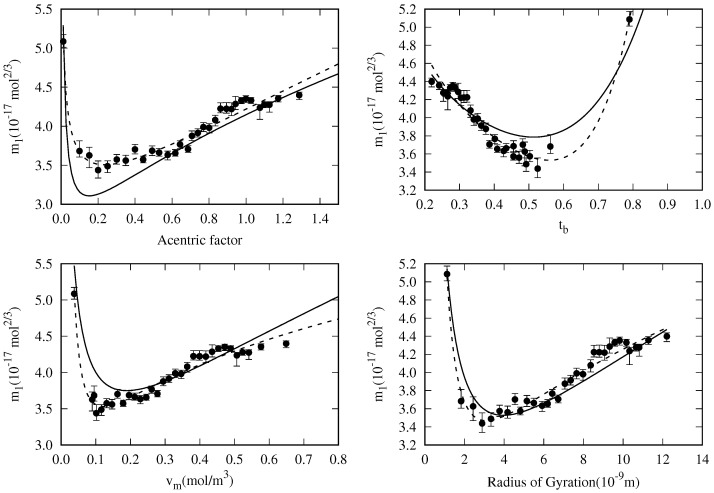
Dependency of $m_{1}$ values with respect some fluid properties (variable *x*). Dashed lines correspond to the least squares fit of the data shown as points to $m_{1}\left(x\right)=a_{1}x^{-n_{1}}+a_{2}x^{n_{2}}$. Solid lines correspond to the same correlation for $m_{1}$ using the coefficients in Table 4.

**Figure 11:**
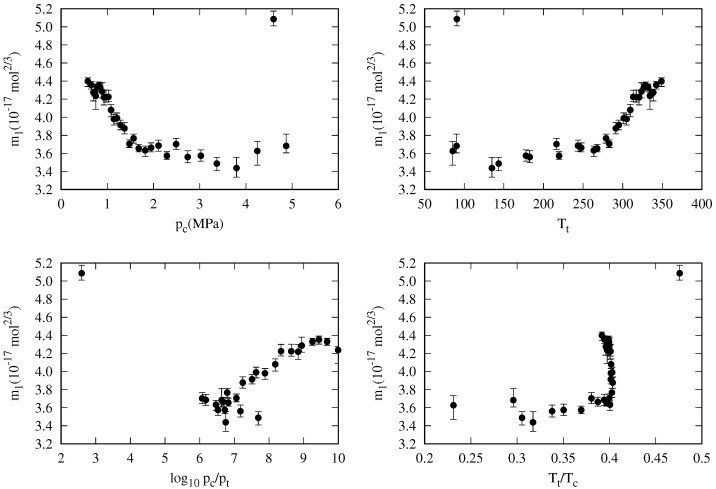
Dependency of $m_{1}$ values from some fluid properties showing that the properties are not good candidates for a correlation.

We will first explore the $m_{1}$ dependence from the fluid properties, as this is the most sensitive coefficient; i.e., a slight deviation in its values leads to a significant increase in the MAPD. When plotting $m_{1}$ versus the fluid properties listed in Table 3, one can see that there are some well-behaved (see Figure 10) and some badly behaved (see Figure 11) candidates for a correlation. Another, as the dipole moment (μ), cannot be used as it has the same value (zero) for all the *n*-alkanes family.

For those properties that are well behaved, the following functional dependency is proposed here:

(32) $$m_{1}\left(x\right)=a_{1}x^{-n_{1}}+a_{2}x^{n_{2}},\;\left(n_{1}>0,n_{2}>0\right),$$

where *x* is the chosen fluid property. In Figure 10, the dashed lines are the fits of the $m_{1}$ values to Equation (32) obtained using a least squares method.

To keep the number of fitting parameters as low as possible, we have considered that $m_{0}$ and $m_{2}$ are constants, and $m_{1}$ is correlated with the chosen fluid property (*x*) as shown in Equation (32). Then, the number of fitting coefficients for $c_{model}^{*}$ will be six, that is $\left\{m_{0},\overset{m_{1}}{\overset{︷}{a_{1},n_{1},a_{2},n_{2}}},m_{2}\right\}$. The fitting coefficients have been obtained by minimizing the OMAPD_*fit*_ of the surface tension data, defined in Equation (30). The initial values for $m_{0}$ and $m_{2}$ are the ones previously determined in the constant model, whereas the others ($a_{1},n_{1},a_{2},n_{2}$) are the corresponding ones to the functions in dashed lines in Figure 10, which have been obtained with a least squares method to the $m_{1}$ data. The minimization procedure is the same as that used for the specific correlation fitting.

As shown in Figure 10, the dashed lines of the $m_{1}$ fits are in better agreement than the solid lines corresponding to the global fit. This is an expected result, provided that in the global fit, the coefficients $m_{0}$ and $m_{2}$ have been regarded as constant for all the fluids, and a small deviation in the $m_{1}$ correlation is needed to fulfill this requirement.

The error bars shown in Figure 10 represent the variation range of the corresponding $m_{1}$ values to increase the MAPD by 0.25% keeping $m_{0}$ and $m_{2}$ fixed. For example, in the case of the radius of gyration, the figure shows that the disagreement between the general correlation (solid lines) and the $m_{1}$ points should increase the MAPD of each fluid by about 0.5% from the specific correlation (dashed lines).

Table 4 summarizes the coefficients and the statistical deviations of the correlations for the parameter $m_{1}$ when correlating with different physical properties. It can be seen that the best correlation is found when the radius of gyration is used (OMAPD_*fit*_ = 1.78%), followed by $v_{c}$ (OMAPD_*fit*_ = 1.94%) and $v_{m}$ (OMAPD_*fit*_ = 1.99%). When the whole set is considered in evaluating the OMAPD_*whole*_, the deviations are 2.26%, 2.52%, and 2.78%, respectively.

The low deviations obtained when using different well-behaved fluid properties suggest that the analytical form of the reduced influence parameter and the choice of the parameters are sound. Thus, it seems natural to have a fixed parameter ($m_{0}$) governing the high-temperature range, another fixed parameter $m_{2}$ governing the rate of change with the temperature of the surface tension, and other fluid-dependent properties related to the surface tension at the triple point temperature.

Although the deviations found when using different fluid parameters are very similar, it is quite appealing that the three lowest deviations are related to geometrical fluid properties: radius of gyration, critical volume, and liquid molar volume at 298.15 K and 101,325 Pa.

The radius of gyration is related to the molecule’s shape, defined as the distance from the center of mass that a particle with the same mass as the molecule will lead to the same molecule’s momentum of inertia [104]. This property has been used by other authors in the development of correlations for the surface tension of ketones, silanes, and carboxilic acids [114,115,116] or the viscosity of silanes [117]. Then, it can be considered an adequate and useful input property for the correlation of fluid surface tension.

Table 5 shows detailed information on the number of data, fitted or not fitted, and the statistical deviations for each fluid when using the radius of gyration as input property. When considering the whole data set, all the MAPDs are below 4%, with the exception of *n*-butane, and they are below 3% (except for *n*-triacontane) for the fitting set.

Although we have included figures of surface tension and the correlations studied using the radius of gyration for all the n-alkanes in the Supplementary Materials, it is worthwhile to briefly discuss the results obtained for a few selected fluids.

Figure 9 shows that for methane, the general correlation deviates from the surface tension data in the high-temperature range. For ethane, there are two data trends at lower temperatures, and the general correlation follows a different trend than the specific one. It can also be appreciated that the deviations in the high-temperature range are mainly due to the disagreement between data and not to the correlation performance.

**Figure 12:**
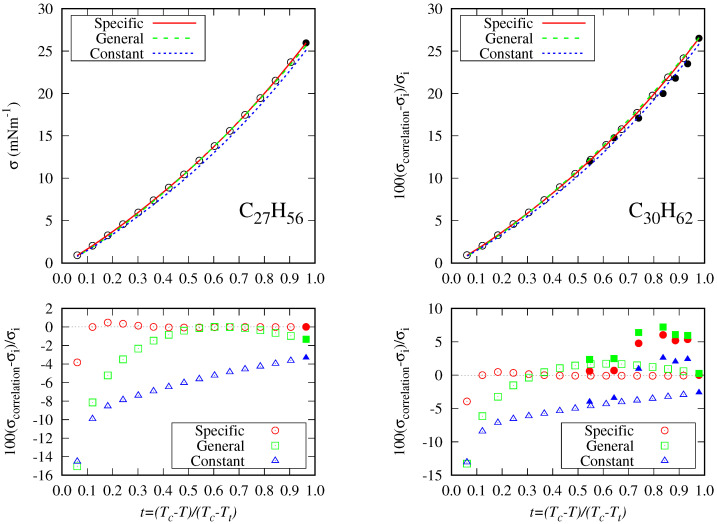
Surface tension data for *n*-heptacosane and *n*-triacontane and percentage deviations from the correlations considered here (lines). The open symbols are the data from Sugden’s correlation included in the DIPPR database. Closed symbols represent the fitting set data.

On the other hand, as an example for those fluids with few data in the fitting set, Figure 12 shows the surface tension data for *n*-heptacosane (one data point) and *n*-triacontane (seven data points). As can be seen, the specific model behaves very well but deviates about 4% at higher temperatures. As expected, the general correlation behaves better than the constant model for both fluids. The data dispersion in the lower temperature range between the DIPPR predicted data and the fitting set can be appreciated in *n*-triacontane.

The second and third lowest OMAPD values are obtained when the critical and molar volumes are used as input properties. Nevertheless, the use of the molar volume rather than the critical one is recommended because it is more easily accessible from the experimental point of view.

The next input parameter with low OMAPD values is the reduced normal boiling temperature. Detailed information (similar to that in Table 5) for the readers interested in the performance of these fluid properties have been included in the Supplementary Materials.

Finally, in Table 4, the statistical deviations obtained when the acentric factor is used as a correlating property are listed. The advantage of using this parameter is that only the information of the triple point temperature ($T_{t}$) is added to the information required for the PR78 EoS ($T_{c}$, $p_{c}$, and ω). The obtained OMAPD of 2.57% of the global fit only increases by 0.31% the value obtained when using the radius of gyration, and the mean deviation of the whole set is the lowest one compiled in Table 4. Detailed statistical information is included in the Supplementary Materials.

## 6. Conclusions

In this paper, the data available for the surface tension of *n*-alkanes have been compiled and adequately selected. In particular, 2681 data points were selected for 32 fluids, and the number of data points selected for each fluid ranged from 15 to 363. The available surface tension data show a high dispersion in some cases, as the values for the same fluid and temperature from different sources are usually in apparent disagreement. This can influence the results obtained when these data are used in a fitting procedure.

The Peng–Robinson-78 EoS has been selected to obtain the equilibrium vapor and liquid densities, and these properties were used as inputs in calculating the influence parameter according to the Density Gradient Theory. In particular, values for the reduced influence parameter were obtained for every fluid and temperature at which selected data for the surface tension were available.

A new analytical expression containing three adjustable coefficients ($m_{0}$, $m_{1}$, and $m_{2}$) and a fixed exponent (n=−0.392) is proposed to fit the reduced influence parameter values versus the reduced temperature. This correlation requires each fluid’s critical and triple point temperatures as input properties. Also, the analytical expression proposed holds the Miqueu et al. ($m_{0}=0$) and Zuo and Stenby ($m_{1}=m_{0}$, $m_{2}=0$, *n* adjustable) correlations as particular cases.

The correlation coefficients are related to the reduced influence parameter: $m_{0}$ determines the behavior near the critical point temperature, $m_{1}$ is the value at the triple point temperature, and $m_{2}$ is the slope at the triple point temperature. The new proposal’s performance is comparable to the Guggenheim–Katayama correlation reported by other authors, so it can be considered an alternative.

From the analysis of the 96 fitting coefficients for all the fluids, it is observed that $m_{0}$ and $m_{2}$ can be fixed, and $m_{1}$ can be correlated with some fluid property, such as radius of gyration, molar volume, acentric factor, etc. Thus, a four-coefficient analytical expression for $m_{1}$ is proposed and fitted using the t≥0.02 data without considering DIPPR data using Sugden’s correlation. The results show that the best results are obtained when the radius of gyration is used as an input parameter, and the results using critical point volume, molar volume, and other parameters are also good alternatives.

A new general correlation, containing six adjustable coefficients, can be used as a predictive tool to populate those temperature ranges for which there are no available data.

New studies will focus on the role of $m_{0}$ and $m_{2}$ in addition to the radius of gyration as an input property for other fluid families. Also, the extension of the proposed correlation to mixtures containing *n*-alkanes will be evaluated in future works.

## Figures and Tables

**Table 1:** Fitting parameters $m_{0}$, $m_{1}$, and $m_{2}$ of Equation (20), number of available data (*N*) and data fitted ($N_{fit}$) when $N\neq N_{fit}$. Mean absolute percentage deviation (MAPD), mean deviation (MD), maximum percentage deviation (PD_m_), and reduced temperature at which the PDm is found for the fluids studied. NC means number of carbons. For the fluids **in boldface**, there are not enough data in the high temperature range, so the value $m_{0}=0$ is obtained. The number between brackets indicates the variation in the last digits of the parameter value (positive or negative) that increases the MAPD by 0.25% when the others parameters are kept fixed. For instance, 0.0(81+) in parameter $m_{0}$ means that if one takes the value 8.1 instead of 0.0, the obtained MAPD would be 0.25% greater than the MAPD with $m_{1}=0.0$.

CN	Fluid	$m_{0}$	$m_{1}$	$m_{2}$	$N/N_{\mathrm{fit}}$	MAPD	MD	PD_m_	$t_{{\mathrm{PD}}_{m}}$
		$(10^{-17}$ mol^2/3^)	$(10^{-17}$ mol^2/3^)	$(10^{-17}$ mol^2/3^)		(%)	(%)	(%)	
1	methane	7.5(12^+^)	5.086(88^+^)	−3.35(34^+^)	127/126	0.97	−0.28	8.5	0.11
2	ethane	5.1(10^+^)	3.68(13^+^)	−3.63(31^+^)	163/160	2.06	−0.77	19.2	0.01
3	propane	4.92(48^−^)	3.63(16^−^)	−3.08(24^−^)	193/191	1.80	0.04	27.2	0.01
4	*n*-butane	3.70(51^+^)	3.44(12^+^)	−3.75(23^−^)	126/118	3.14	1.17	48.9	0.01
5	*n*-pentane	3.84(76^−^)	3.488(80^−^)	−3.25(21^−^)	149/143	1.72	1.03	36.5	0.01
6	*n*-hexane	3.8(14^−^)	3.574(64^+^)	−3.18(17^+^)	270/269	0.97	0.18	12.8	0.07
7	*n*-heptane	4.32(74^+^)	3.560(68^+^)	−2.78(20^+^)	363/357	0.89	0.09	9.7	0.22
8	*n*-octane	5.5(20^−^)	3.702(65^+^)	−2.55(23^+^)	196/194	1.04	0.43	41.1	0.01
9	***n*** **-nonane**	0.0(81^+^)	3.573(44^+^)	−2.86(21^+^)	78	0.41	0.07	3.0	0.80
10	*n*-decane	3.8(26^−^)	3.686(62^−^)	−2.16(27^+^)	149	0.95	−0.08	5.8	0.68
11	***n*** **-undecane**	0.0(34^+^)	3.663(53^+^)	−2.25(27^+^)	60	0.55	0.05	2.6	0.49
12	*n*-dodecane	5.2(38^+^)	3.633(65^−^)	−2.09(33^−^)	100	0.91	0.30	6.7	0.91
13	*n*-tridecane	4(12^+^)	3.655(45^+^)	−2.22(28^+^)	48	0.33	−0.02	1.3	0.69
14	*n*-tetradecane	15(14^−^)	3.767(53^−^)	−1.85(44^+^)	49	0.49	0.07	1.9	0.97
15	*n*-pentadecane	12(44^+^)	3.705(44^+^)	−1.97(47^+^)	40	0.40	−0.01	2.0	0.67
16	*n*-hexadecane	7.7(37^−^)	3.876(64^+^)	−2.19(67^−^)	127	1.33	0.12	6.8	0.40
17	*n*-heptadecane	7.3(11^−^)	3.914(54^+^)	−2.46(27^−^)	44	0.44	0.08	2.1	0.78
18	*n*-octadecane	8.0(13^+^)	3.990(59^−^)	−2.36(30^−^)	39	0.48	0.05	1.9	0.79
19	*n*-nonadecane	8.13(79^−^)	3.981(67^−^)	−2.48(24^−^)	23	0.70	0.02	5.4	0.34
20	*n*-eicosane	7.7(11^+^)	4.079(75^−^)	−2.78(26^−^)	38	1.04	−0.10	11.7	0.33
21	*n*-heneicosane	8.0(10^+^)	4.225(76^+^)	−2.83(22^+^)	28	0.47	−0.18	2.8	0.07
22	*n*-docosane	7.8(12^+^)	4.223(78^+^)	−2.75(31^+^)	32	0.58	−0.28	2.9	0.07
23	*n*-tricosane	7.8(12^+^)	4.219(84^−^)	−2.84(27^+^)	36	0.98	0.12	3.4	0.06
24	*n*-tetracosane	7.9(13^+^)	4.286(96^+^)	−2.79(31^+^)	36	0.93	−0.11	3.8	0.98
25	*n*-pentacosane	8.17(67^+^)	4.330(40^−^)	−2.54(10^−^)	15	0.29	−0.21	3.4	0.06
26	*n*-hexacosane	8.2(11^+^)	4.352(49^−^)	−2.43(19^−^)	31	0.30	−0.04	3.7	0.06
27	*n*-heptacosane	8.07(60^+^)	4.331(41^−^)	−2.42(12^−^)	16	0.31	−0.19	3.8	0.06
28	*n*-octacosane	7.80(97^+^)	4.24(15^−^)	−2.35(25^−^)	24	1.67	1.26	7.7	0.78
29	*n*-nonacosane	7.70(58^+^)	4.278(42^−^)	−2.33(12^−^)	16	0.31	−0.18	3.9	0.06
30	*n*-triacontane	7.72(83^+^)	4.276(95^−^)	−2.32(19^−^)	22	1.29	0.86	6.0	0.84
32	*n*-dotriacontane	7.58(90^+^)	4.356(45^−^)	−2.24(17^−^)	25	0.32	−0.09	4.0	0.06
36	*n*-hexatriacontane	7.65(58^+^)	4.399(58^−^)	−2.15(15^−^)	18	0.43	−0.13	4.6	0.06
	Overall mean absolute percentage deviation (defined in Equation (30))					0.79			

**Table 2:** Statistical figures of the general correlation when all the $m_{i}$ are taken as constants (no fluid dependence). CN is the carbon number, *N* the number of data, MAPD the mean absolute percentage deviation, MD the mean deviation, PD_m_ the maximum absolute percentage deviation, and $t_{\mathrm{PDm}}$ is the reduced temperature of the maximum percentage deviation. The subscript fit is added for those figures calculated with the fitting set (2427 values). The other results are for the whole data set (2681 values). The fitting parameters are $m_{0}=5.983$, $m_{1}=4.060$, and $m_{2}=-1.810$ (in $10^{-17}$ mol^2/3^ units). Maximum absolute values for MAPD, MD, and PD_m_ are written in **boldface**.

CN	$N/N_{\mathrm{fit}}$	MAPD/MAPD_*fit*_	MD/MD_*fit*_	PD_m_/PD_m*fit*_	$t_{\mathrm{PDm}}/t_{{\mathrm{PDm}}_{\mathrm{fit}}}$
		(%)	(%)	(%)	
1	127/126	**12.99** **/13.05**	**−12.99**/**−13.05**	20.95/20.95	0.11/0.11
2	163/160	3.48/3.28	−2.33/−2.29	15.80/14.41	0.01/0.02
3	193/191	2.14/2.00	−0.49/−0.34	22.59/**22.10**	0.01/0.06
4	126/118	5.42/2.59	2.35/−0.70	**75.48**/15.04	0.01/0.03
5	149/143	3.53/2.06	3.00/1.51	62.01/17.12	0.01/0.02
6	270/269	1.82/1.76	1.20/1.14	17.35/16.20	0.01/0.04
7	363/357	2.94/2.71	2.90/2.68	20.39/17.97	0.01/0.05
8	196/194	2.41/2.00	2.17/1.76	45.07/5.72	0.01/1.00
9	78	3.52	3.52	6.93	0.99
10	149	3.80	3.74	9.71	0.12
11	60	4.36	4.36	10.43	0.30
12	100	4.98	4.98	12.18	0.91
13	48	4.30	4.30	5.86	0.99
14	49	3.60	3.39	5.72	0.97
15	40	4.19	4.19	5.37	0.95
16	127/117	2.40/2.31	1.52/1.90	7.31/7.17	0.08/1.00
17	44/34	1.56/1.06	−0.13/0.80	7.16/2.15	0.07/0.78
18	39/29	1.57/0.55	−0.92/0.34	9.37/1.40	0.07/1.00
19	23/12	2.85/1.20	−2.49/−0.51	10.41/10.20	0.07/0.34
20	38/25	2.89/1.43	−2.78/−1.26	17.69/17.69	0.33/0.33
21	28/14	5.86/4.78	−5.86/−4.78	14.00/7.35	0.07/0.49
22	32/19	5.16/4.05	−5.16/−4.05	13.18/6.99	0.07/0.54
23	36/22	5.05/3.74	−5.05/−3.74	13.93/6.97	0.06/0.52
24	36/22	5.60/4.43	−5.56/−4.36	14.37/7.22	0.06/0.61
25	15/ 1	6.92/3.48	−6.92/−3.48	14.70/3.48	0.06/0.95
26	31/19	5.40/3.96	−5.40/−3.96	14.97/4.45	0.06/0.81
27	16/ 1	6.45/3.32	−6.45/−3.32	14.52/3.32	0.06/0.96
28	24/ 9	4.42/2.57	−3.35/0.29	13.13/4.23	0.06/0.78
29	16/ 1	5.41/2.60	−5.41/−2.60	12.95/2.60	0.06/0.97
30	22/ 7	4.64/2.57	−3.91/−0.30	13.07/4.01	0.06/0.55
32	25/12	4.92/3.64	−4.92/−3.64	12.61/4.23	0.06/0.85
36	18/ 1	5.67/3.48	−5.67/−3.48	13.36/3.48	0.06/1.00
-	2681/2427			75.48/22.10	
	$N_{fluid}$	OMAPD/	OMD/		
		OMAPD_*fit*_	OMD_*fit*_		
	32	4.38/3.35	−1.38/−0.53		

**Table 3:** Fluid properties considered in the possible $m_{i}$ correlations.

	Name	Symbol	Units
0	No dependency (constant)	-	-
1	Critical pressure	$p_{c}$	Pa
2	Critical temperature	$T_{c}$	K
3	Acentric factor	ω	-
4	Critical compressibility factor	$Z_{c}$	-
5	Critical volume	$v_{c}$	L mol^−1^
6	Melting temperature	$T_{f}$	K
7	Triple point temperature	$T_{t}$	K
8	Normal boiling point temperature	$T_{b}$	K
9	Logarithmic ratio between $p_{c}$ and $p_{t}$	$\log_{10}\left(p_{c}/p_{t}\right)$	-
10	Liquid molar volume at 298.15 K and 101,325 Pa	$v_{m}$	L mol^−1^
11	Radius of gyration	RG	$10^{-9}$ m
12	Dipole moment	μ	Cm
13	Molar weight	$M_{w}$	kg kmol^−1^
14	Reduced triple point temperature	$T_{t}/T_{c}$	-
15	Reduced normal boiling temperature	$T_{b}/T_{c}$	-
16	Pseudo compressibility factor	$p_{c}v_{m}/(RT_{b}$)	-
17	Reduced boiling temperature	$t_{b}=\left(T_{c}-T_{b}\right)/\left(T_{c}-T_{t}\right)$	-

**Table 4:** Adjustable coefficients and statistical deviations for different physical properties considered in the correlation for $m_{1}=a_{1}x^{-n_{1}}+a_{2}x^{n_{2}}$, where *x* is the chosen property.

Adjustable Coefficients		RG	$v_{c}$	$v_{m}$	$t_{b}$	ω
$m_{0}$	($10^{-17}\;{\mathrm{mol}}^{2/3}$)	5.01227	4.91156	5.37325	6.48294	4.99955
$a_{1}$	($10^{-17}\;{\mathrm{mol}}^{2/3}$)	4.40431	1.09159	0.662073	3.03929	0.625431
$n_{1}$		0.885059	0.53364	0.595946	0.254804	0.452929
$a_{2}$	($10^{-17}\;{\mathrm{mol}}^{2/3}$)	1.08825	2.68771	4.84453	5.0875	3.52179
$n_{2}$		0.519495	0.401163	0.542305	4.98139	0.405698
$m_{2}$	($10^{-17}\;{\mathrm{mol}}^{2/3}$)	−2.92951	−2.6442	−2.05972	−2.20715	−3.45186
Statistical figures for the fitting set						
OMAPD_*fit*_ (%)		1.78	1.94	1.99	2.16	2.18
MD_*fit*_ (%)		0.04	−0.02	−0.13	0.16	0.24
PDm_*fit*_ (%)		23.93	24.37	23.76	21.44	24.35
Statistical figures for the whole set						
OMAPD (%)		2.26	2.52	2.78	2.68	2.57
MD (%)		−0.45	−0.79	−1.20	−0.37	0.11
PDm (%)		65.02	63.50	68.36	81.76	64.95

**Table 5:** Statistical figures of the global correlation when $m_{0}=5.012$, $m_{2}=-2.92951$ and $m_{1}=4.40431x^{-0.8851}+1.08825x^{0.5195}$ (all in $10^{-17}$ mol^2/3^ units), with *x* being the radius of gyration. CN is the carbon number, *N* the number of data, MAPD the mean absolute percentage deviation, MD the mean deviation, PD_m_ the maximum absolute percentage deviation, and $t_{\mathrm{PDm}}$ is the reduced temperature of the maximum percentage deviation. The subscript fit is added for those figures calculated with the fitting set. Maximum absolute values for MAPD, MD, and PD_m_ are written in **boldface**.

CN	$N/N_{\mathrm{fit}}$	MAPD/MAPD_*fit*_	MD/MD_*fit*_	PD_m_/PD_m*fit*_	$t_{\mathrm{PDm}}/t_{{\mathrm{PDm}}_{\mathrm{fit}}}$
		(%)	(%)	(%)	
1	127/126	2.49/2.45	−2.20/−2.16	16.28/16.28	0.11/0.11
2	163/160	2.58/2.35	0.09/0.25	20.01/10.71	0.01/0.04
3	193/191	1.84/1.65	0.27/0.48	26.76/**23.93**	0.01/0.06
4	126/118	**4.54**/2.19	1.53/−1.02	**65.02**/10.03	0.01/0.03
5	149/143	2.42/1.25	1.75/0.54	51.44/11.26	0.01/0.02
6	270/269	1.72/1.69	−0.37/−0.41	12.64/12.64	0.07/0.07
7	363/357	1.36/1.22	0.96/0.81	13.96/13.96	0.05/0.05
8	196/194	1.40/1.08	−0.34/−0.68	35.68/4.47	0.01/0.19
9	78	0.54	0.38	3.76	0.54
10	149	1.55	0.97	8.52	0.12
11	60	1.77	1.77	11.63	0.30
12	100	2.37	2.34	7.96	0.91
13	48	1.66	1.66	4.26	0.69
14	49	0.96	0.61	3.76	0.72
15	40	1.67	1.67	4.58	0.74
16	127/117	1.77/1.73	−0.24/−0.09	8.64/7.67	0.08/0.46
17	44/34	1.15/0.84	−0.68/−0.39	8.56/2.20	0.07/0.78
18	39/29	1.50/1.08	−1.24/−0.74	10.54/2.65	0.07/0.99
19	23/12	2.06/1.44	−1.54/−0.64	11.62/7.08	0.07/0.34
20	38/25	2.48/1.97	−2.18/−1.52	14.55/14.55	0.33/0.33
21	28/14	3.80/2.81	−**3.80**/−2.81	14.97/3.63	0.07/0.49
22	32/19	3.15/2.48	−3.15/−2.48	13.98/3.32	0.07/0.63
23	36/22	2.79/1.90	−2.53/−1.47	14.82/3.23	0.06/0.64
24	36/22	3.02/2.20	−2.92/−2.04	15.08/3.94	0.06/0.80
25	15/ 1	3.64/2.25	−3.64/−2.25	15.32/2.25	0.06/0.95
26	31/19	2.32/1.39	−2.31/−1.36	15.59/2.09	0.06/0.93
27	16/ 1	2.53/1.31	−2.53/−1.31	15.04/1.31	0.06/0.96
28	24/ 9	3.33/**4.91**	1.05/**4.91**	13.58/8.81	0.06/0.78
29	16/ 1	2.22/0.07	−0.92/0.07	13.24/0.07	0.06/0.97
30	22/ 7	3.07/4.39	0.83/4.39	13.29/7.22	0.06/0.84
32	25/12	1.64/0.55	−0.11/0.55	12.66/0.97	0.06/0.87
36	18/ 1	2.88/1.30	0.45/1.30	13.31/1.30	0.06/1.00
Overall	2681/2427			65.02/23.93	
	$N_{fluid}$	OMAPD/	OMD/		
		OMAPD_*fit*_	OMD_*fit*_		
	32	2.26/1.78	−0.45/0.04		

## Data Availability

Original data for the surface tension of *n*-alkanes were taken from databases, books, and references, so they are not available here. Graphical displays of the selected values are given in the figures included in the Supplementary Materials.

## Supplementary Materials

The following supporting information can be downloaded at: https://www.mdpi.com/article/10.3390/molecules29235643/s1, Figures S1–S32: Surface tension data and percentage deviations from the considered correlations for the fluids studied; Tables S1–S3: Statistical figures when considering the acentric factor, critical volume, and liquid molar volume as input properties, respectively.
