# Supplementary material for: A New General Correlation for the Influence Parameter in Density Gradient Theory and Peng–Robinson Equation of State for n-Alkanes
Source: Molecules. 2024 Nov 28;29(23):5643. doi: 10.3390/molecules29235643 (PMC11643655; doi:10.3390/molecules29235643)
Supplement: Supplementary file 1 [file molecules-29-05643-s001.zip › molecules-3306083-supplementary.pdf]

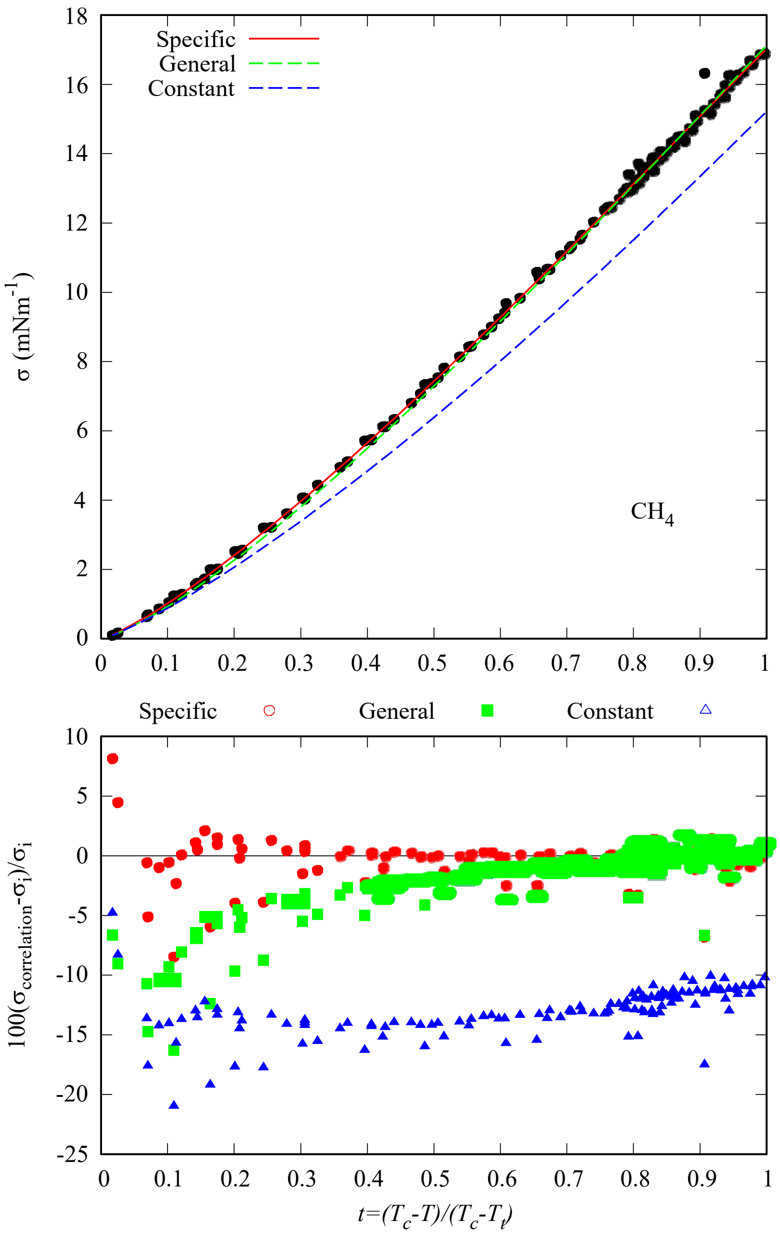

Figure S1. Surface tension data for methane and percentage deviations from the correlations considered here (lines). The open symbols are the data from Sugden's correlation included in the DIPPR database. Closed symbols represent the fitting set data.

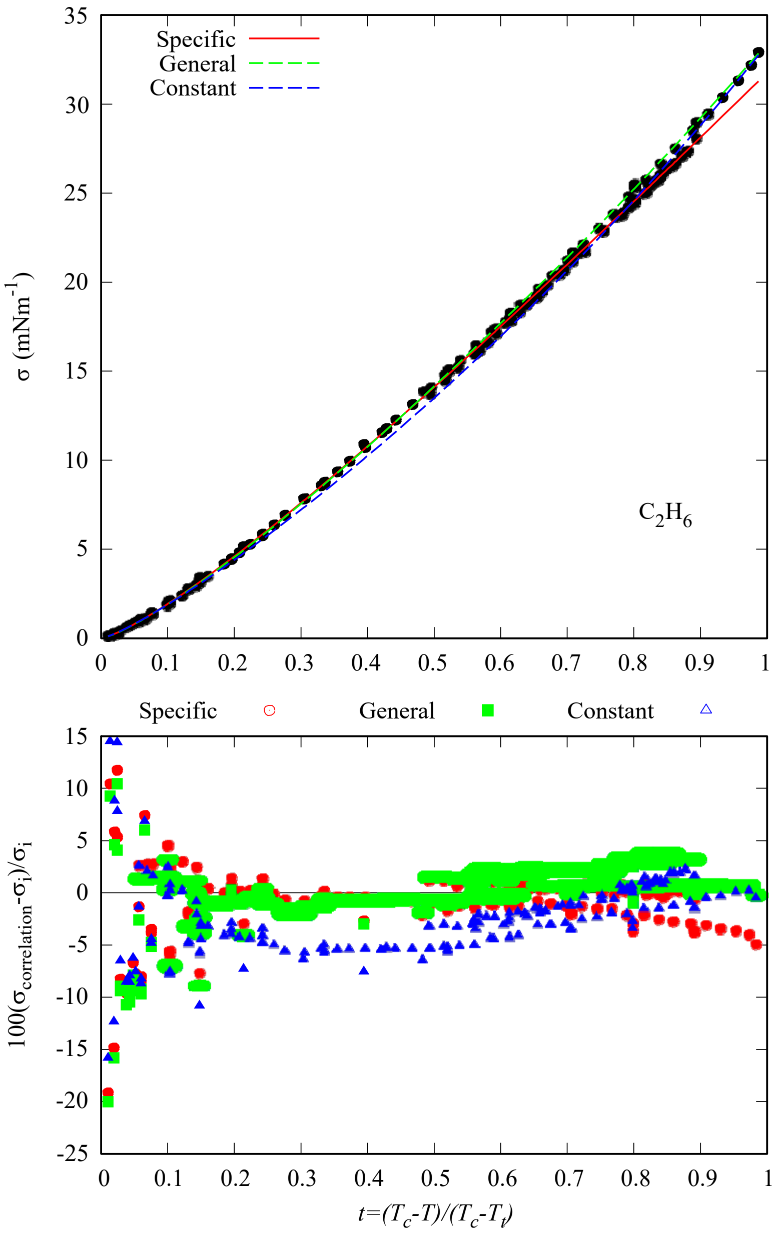

Figure S2. Surface tension data for ethane and percentage deviations from the correlations considered here (lines). The open symbols are the data from Sugden's correlation included in the DIPPR database. Closed symbols represent the fitting set data.

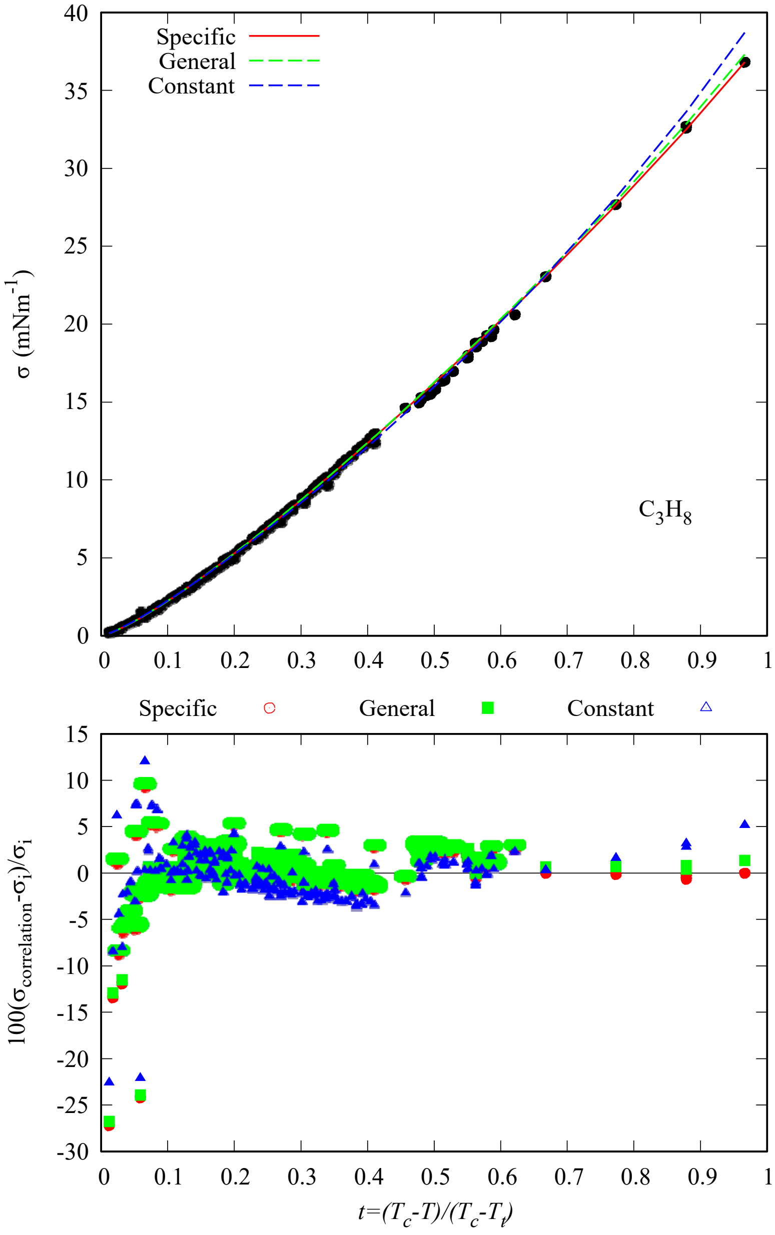

Figure S3. Surface tension data for propane and percentage deviations from the correlations considered here (lines). The open symbols are the data from Sugden's correlation included in the DIPPR database. Closed symbols represent the fitting set data.

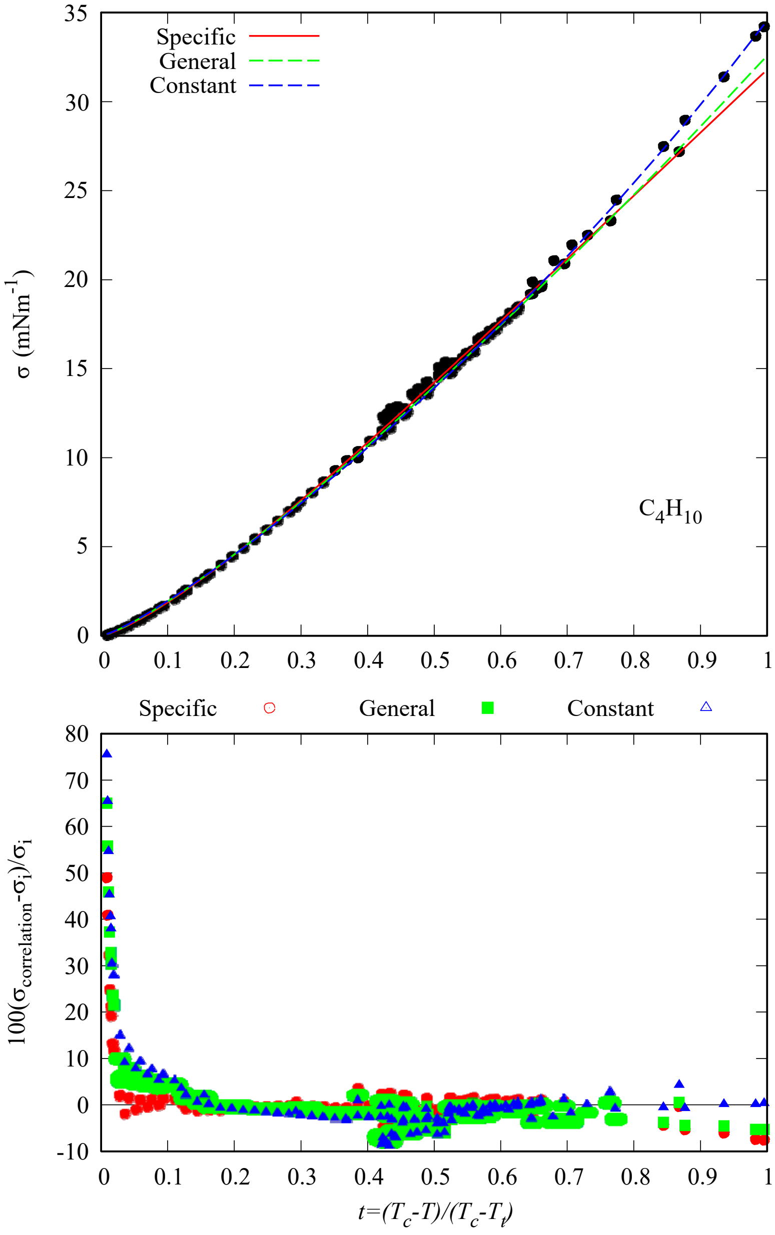

Figure S4. Surface tension data for *n*-butane and percentage deviations from the correlations considered here (lines). The open symbols are the data from Sugden's correlation included in the DIPPR database. Closed symbols represent the fitting set data.

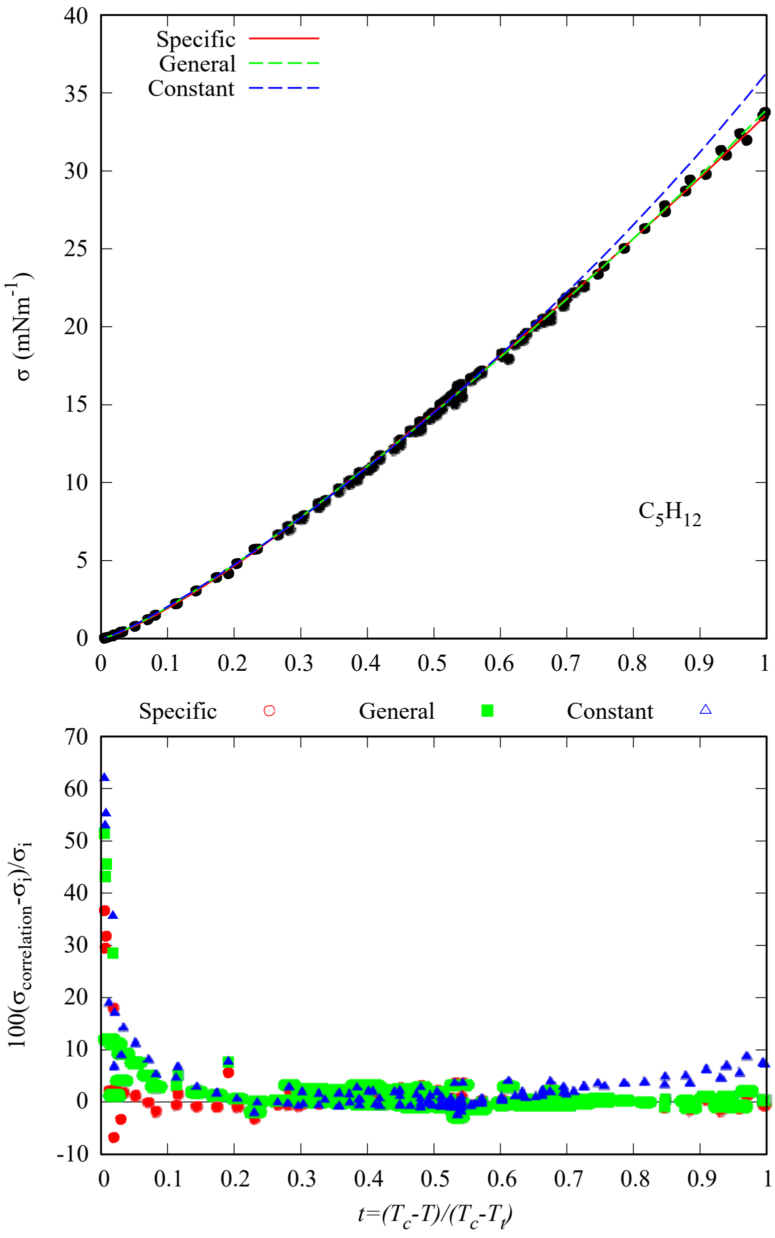

Figure S5. Surface tension data for *n*-pentane and percentage deviations from the correlations considered here (lines). The open symbols are the data from Sugden's correlation included in the DIPPR database. Closed symbols represent the fitting set data.

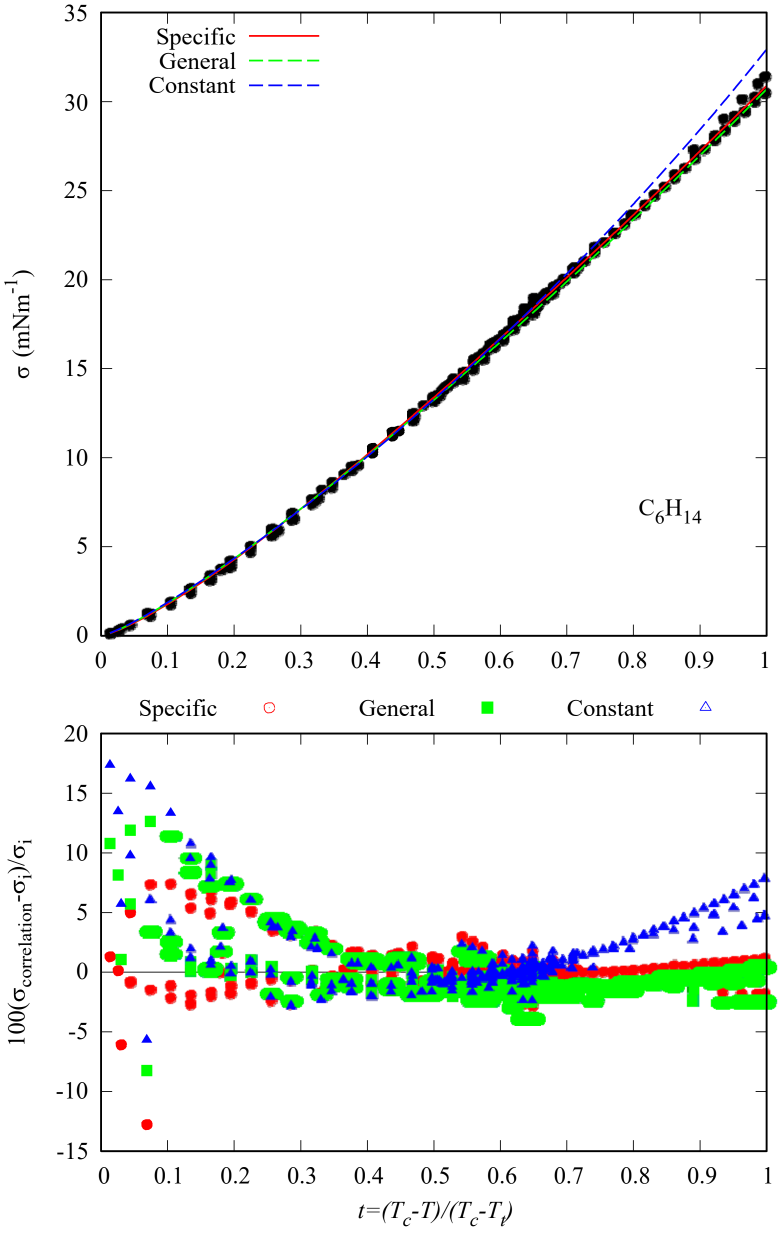

Figure S6. Surface tension data for *n*-hexane and percentage deviations from the correlations considered here (lines). The open symbols are the data from Sugden's correlation included in the DIPPR database. Closed symbols represent the fitting set data.

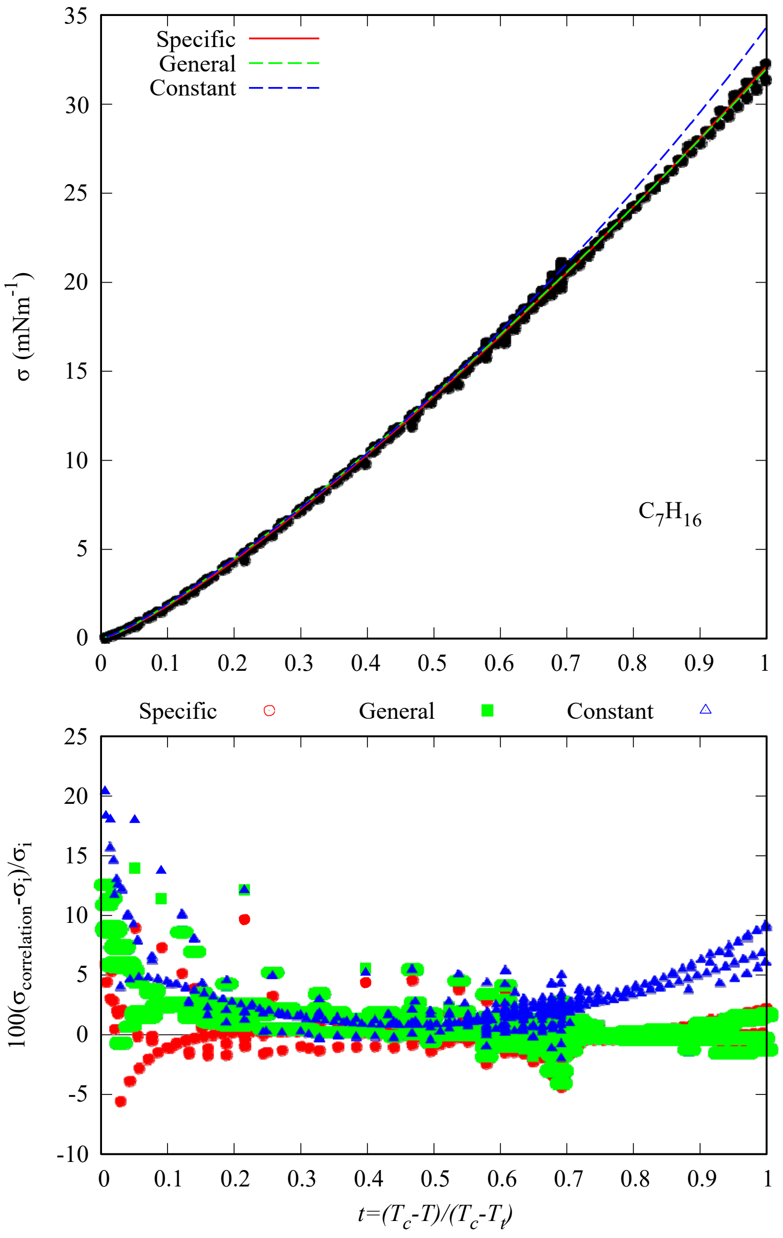

Figure S7. Surface tension data for *n*-heptane and percentage deviations from the correlations considered here (lines). The open symbols are the data from Sugden's correlation included in the DIPPR database. Closed symbols represent the fitting set data.

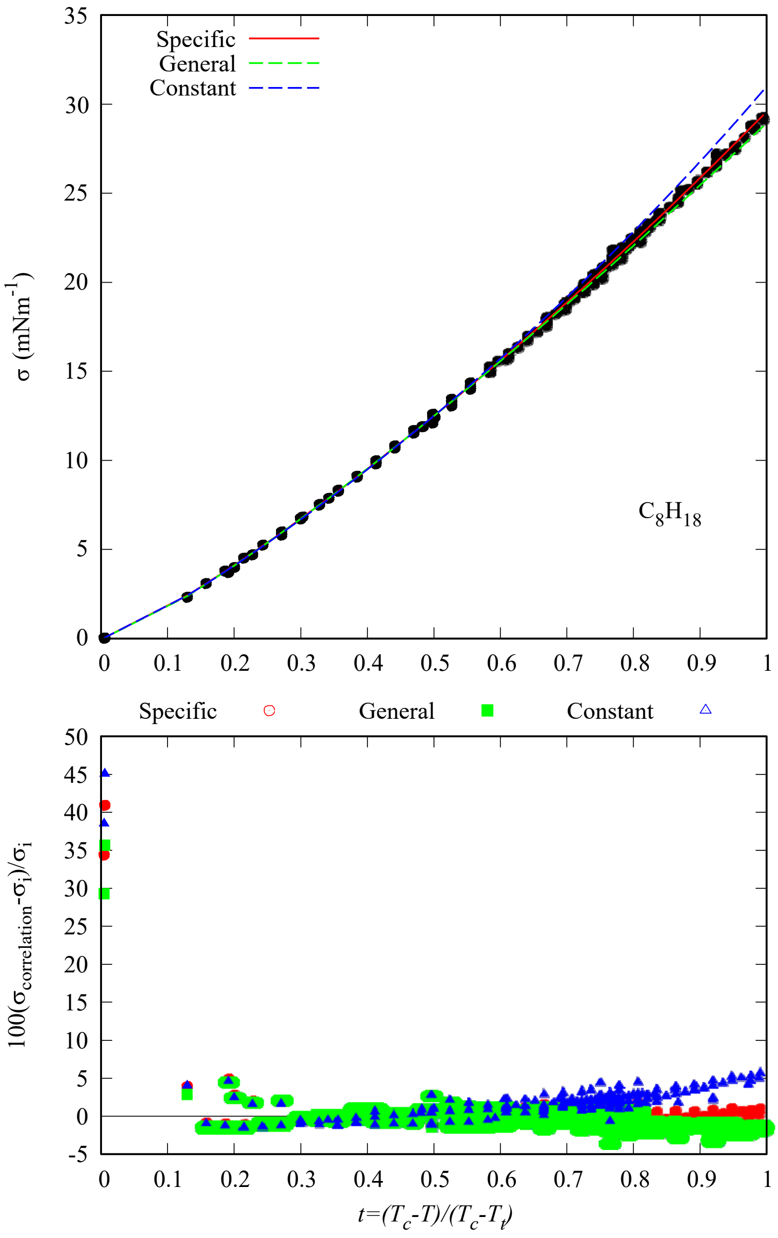

Figure S8. Surface tension data for *n*-octane and percentage deviations from the correlations considered here (lines). The open symbols are the data from Sugden's correlation included in the DIPPR database. Closed symbols represent the fitting set data.

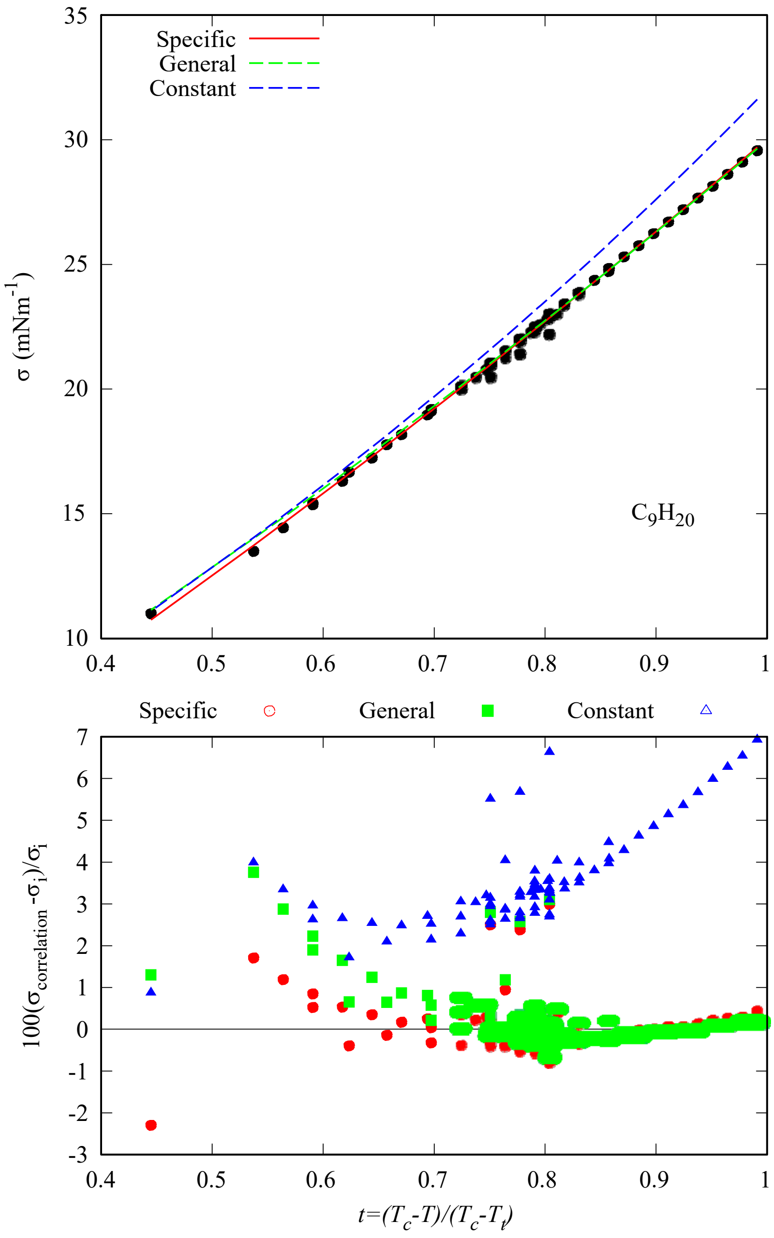

Figure S9. Surface tension data for *n*-nonane and percentage deviations from the correlations considered here (lines). The open symbols are the data from Sugden's correlation included in the DIPPR database. Closed symbols represent the fitting set data.

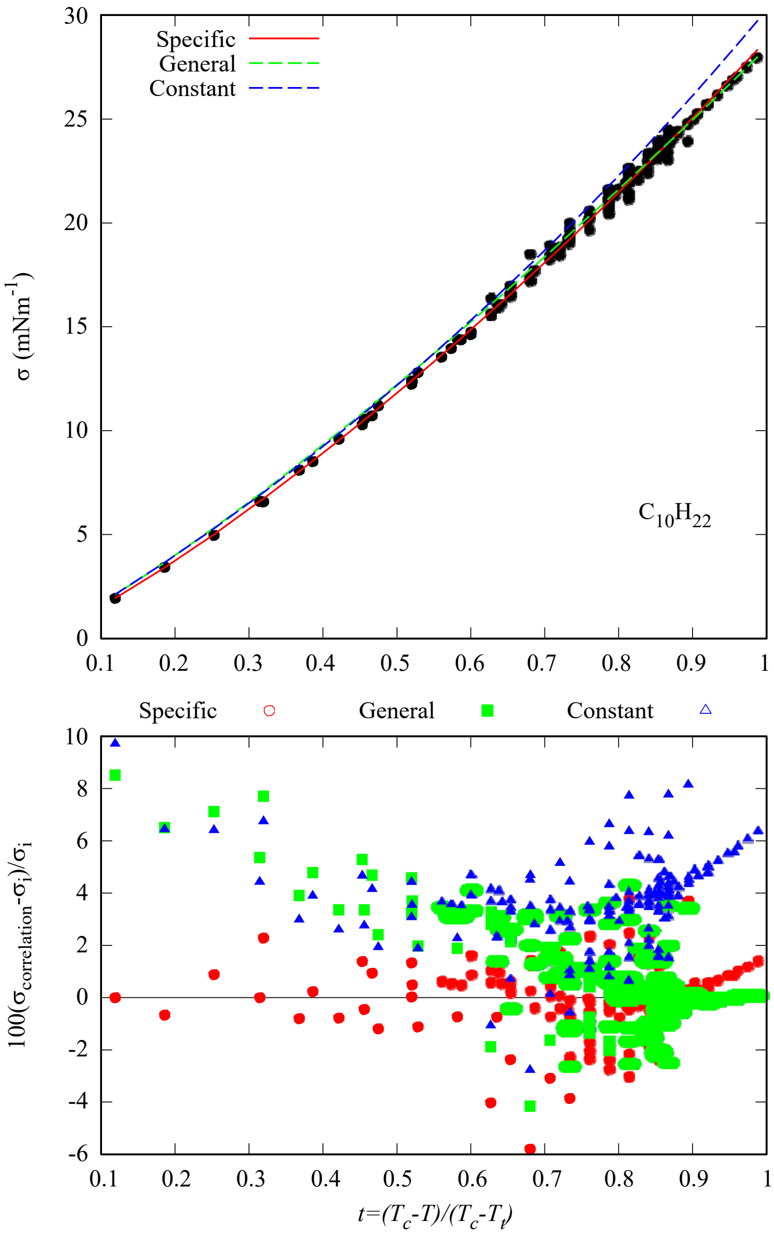

Figure S10. Surface tension data for *n*-decane and percentage deviations from the correlations considered here (lines). The open symbols are the data from Sugden's correlation included in the DIPPR database. Closed symbols represent the fitting set data.

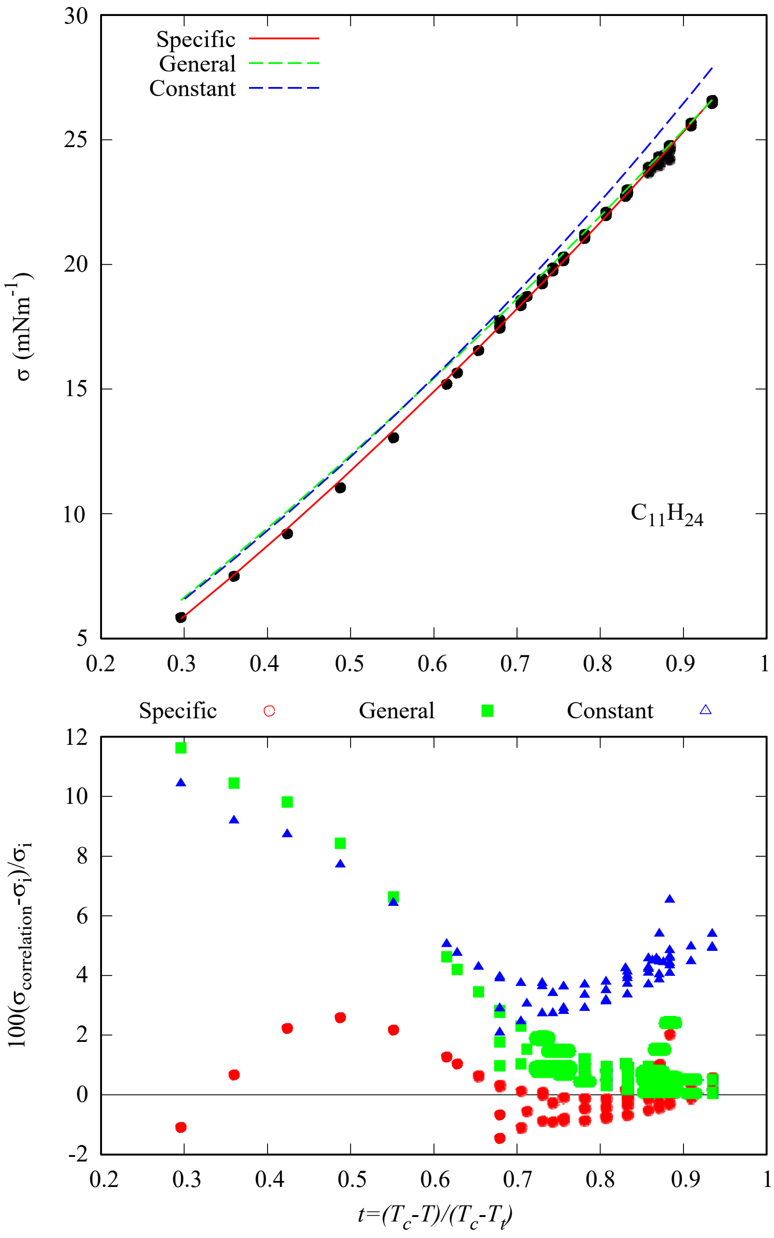

Figure S11. Surface tension data for *n*-undecane and percentage deviations from the correlations considered here (lines). The open symbols are the data from Sugden's correlation included in the DIPPR database. Closed symbols represent the fitting set data.

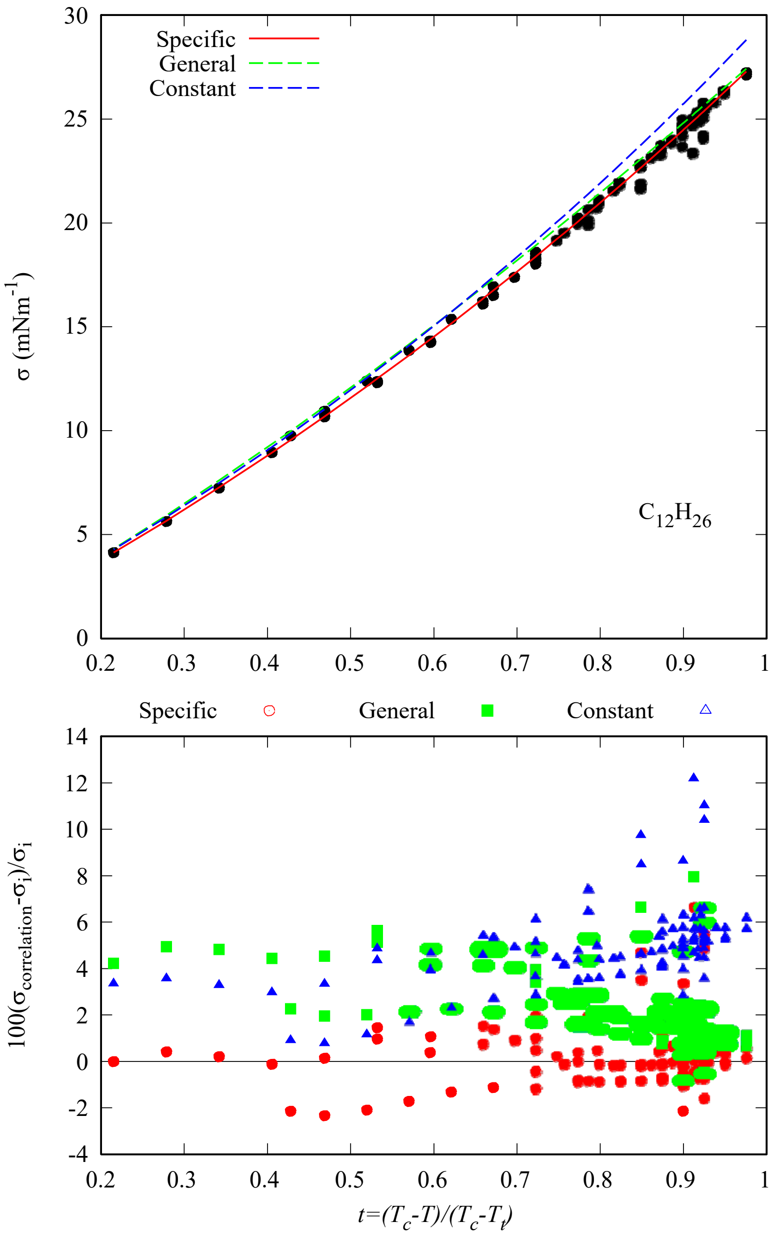

Figure S12. Surface tension data for *n*-dodecane and percentage deviations from the correlations considered here (lines). The open symbols are the data from Sugden's correlation included in the DIPPR database. Closed symbols represent the fitting set data.

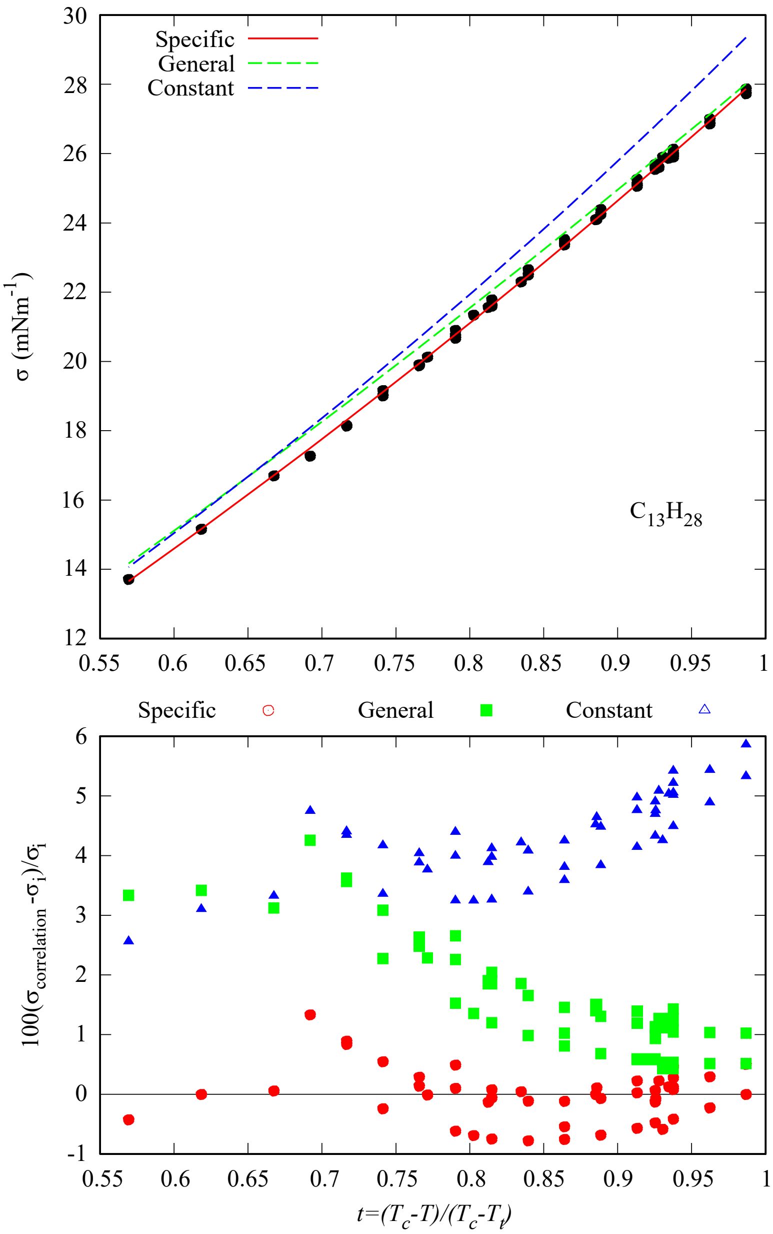

Figure S13. Surface tension data for *n*-tridecane and percentage deviations from the correlations considered here (lines). The open symbols are the data from Sugden's correlation included in the DIPPR database. Closed symbols represent the fitting set data.

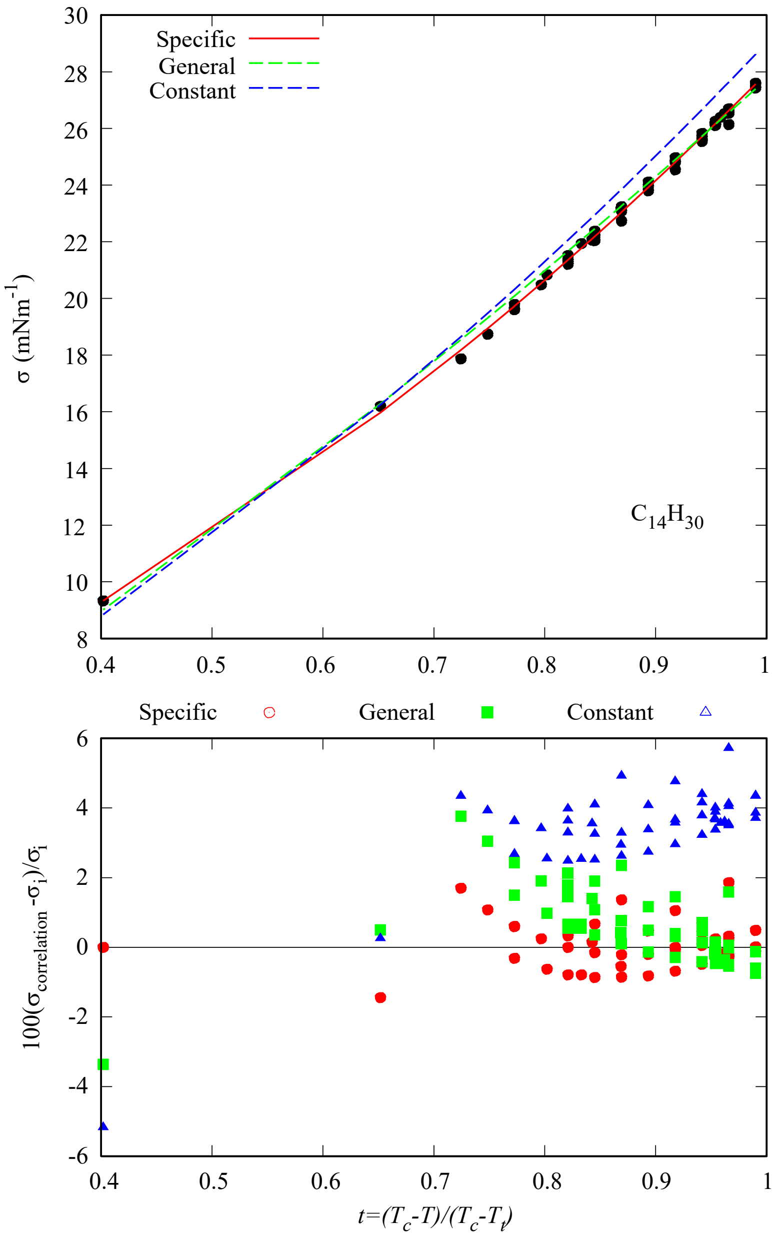

Figure S14. Surface tension data for *n*-tetradecane and percentage deviations from the correlations considered here (lines). The open symbols are the data from Sugden's correlation included in the DIPPR database. Closed symbols represent the fitting set data.

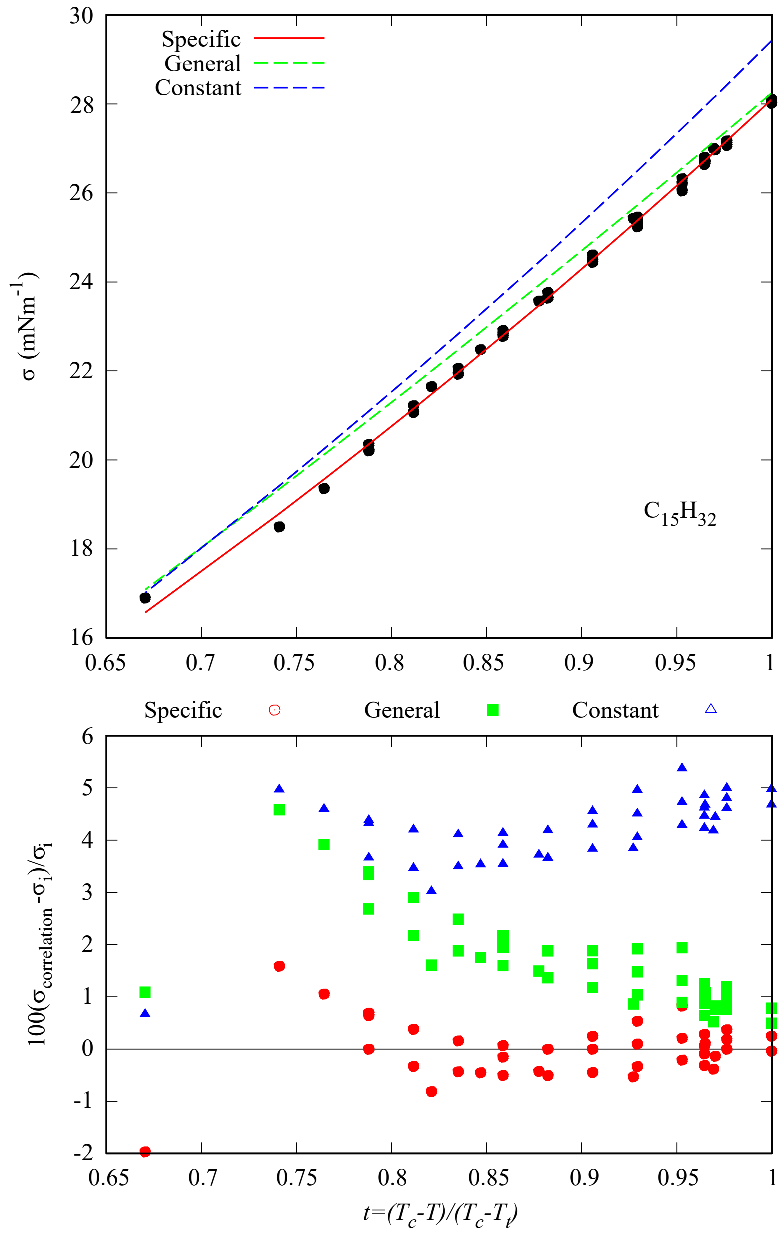

Figure S15. Surface tension data for *n*-pentadecane and percentage deviations from the correlations considered here (lines). The open symbols are the data from Sugden's correlation included in the DIPPR database. Closed symbols represent the fitting set data.

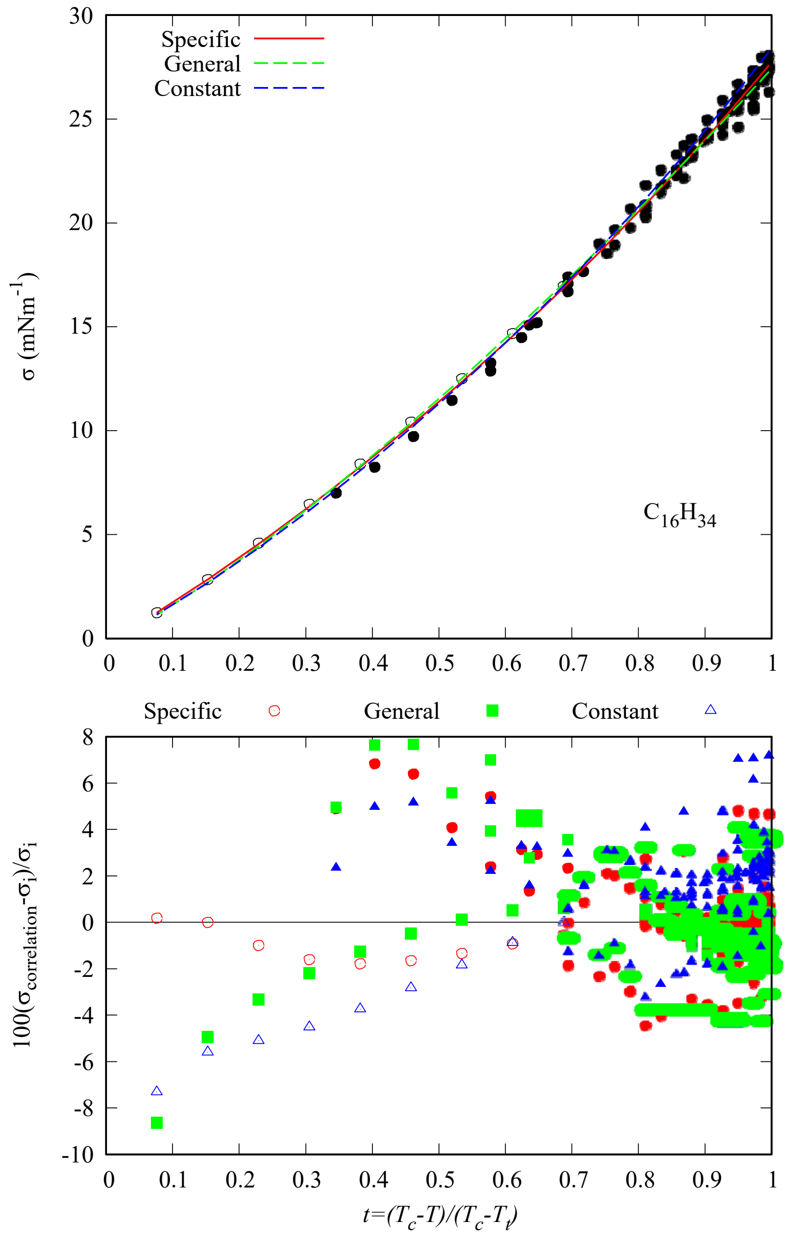

Figure S16. Surface tension data for *n*-hexadecane and percentage deviations from the correlations considered here (lines). The open symbols are the data from Sugden's correlation included in the DIPPR database. Closed symbols represent the fitting set data.

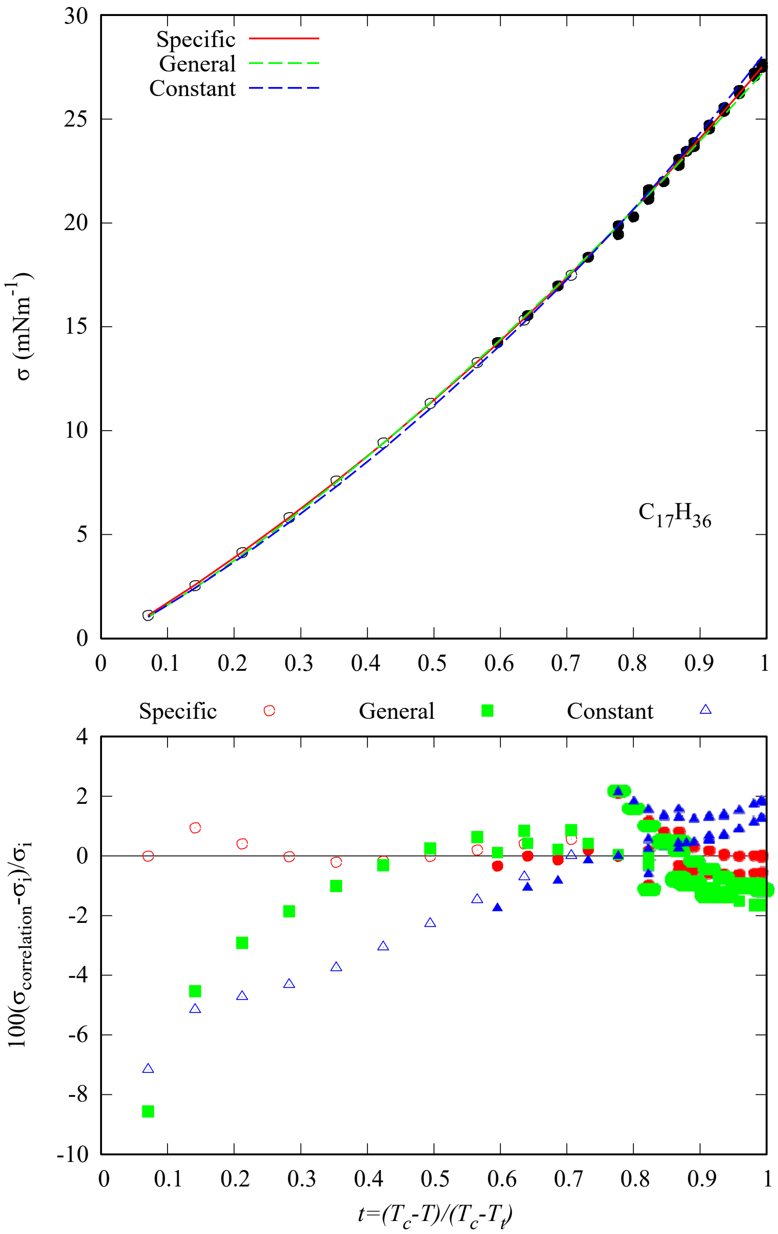

Figure S17. Surface tension data for *n*-heptadecane and percentage deviations from the correlations considered here (lines). The open symbols are the data from Sugden's correlation included in the DIPPR database. Closed symbols represent the fitting set data.

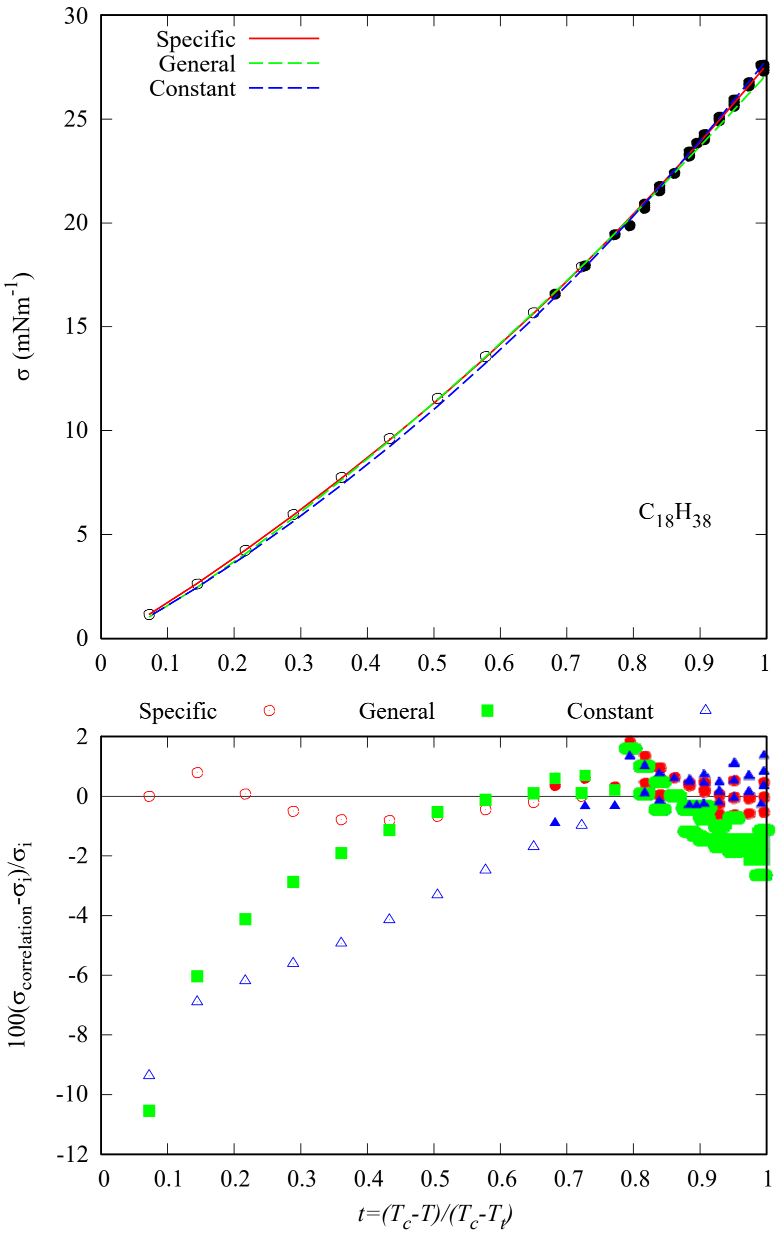

Figure S18. Surface tension data for *n*-octadecane and percentage deviations from the correlations considered here (lines). The open symbols are the data from Sugden's correlation included in the DIPPR database. Closed symbols represent the fitting set data.

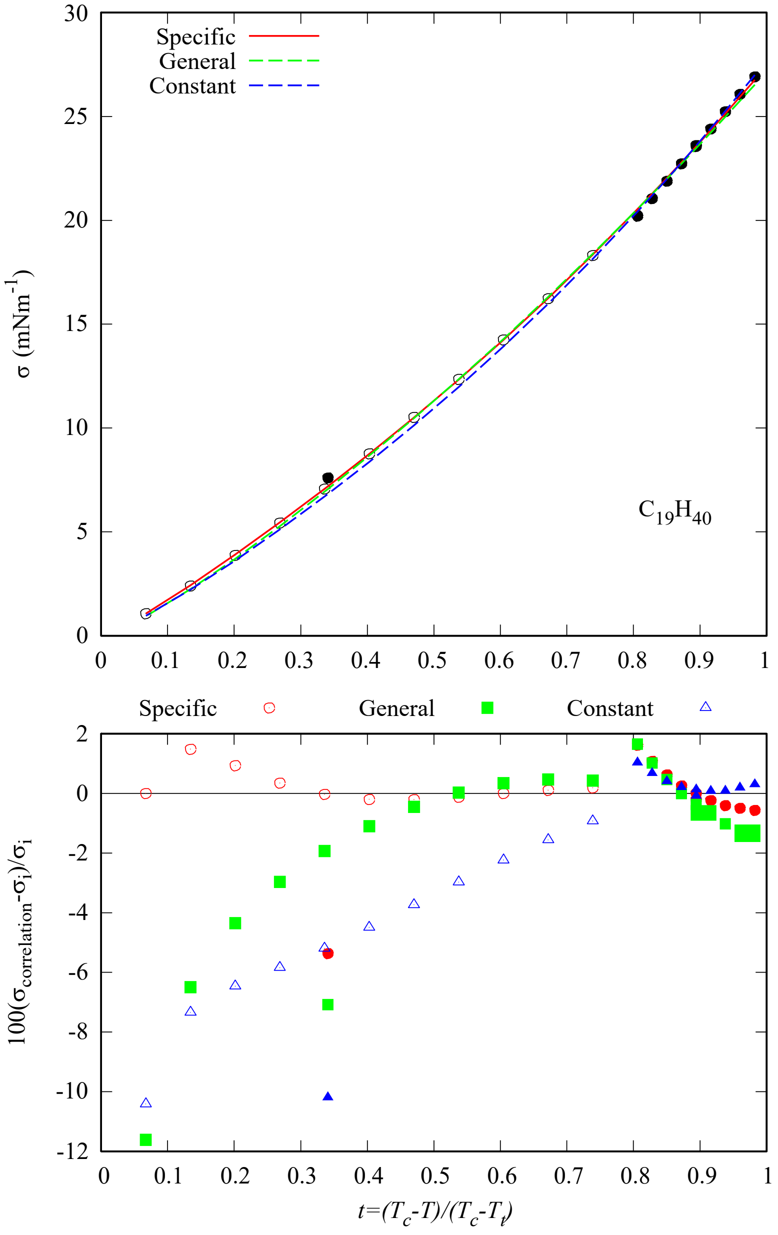

Figure S19. Surface tension data for *n*-nonadecane and percentage deviations from the correlations considered here (lines). The open symbols are the data from Sugden's correlation included in the DIPPR database. Closed symbols represent the fitting set data.

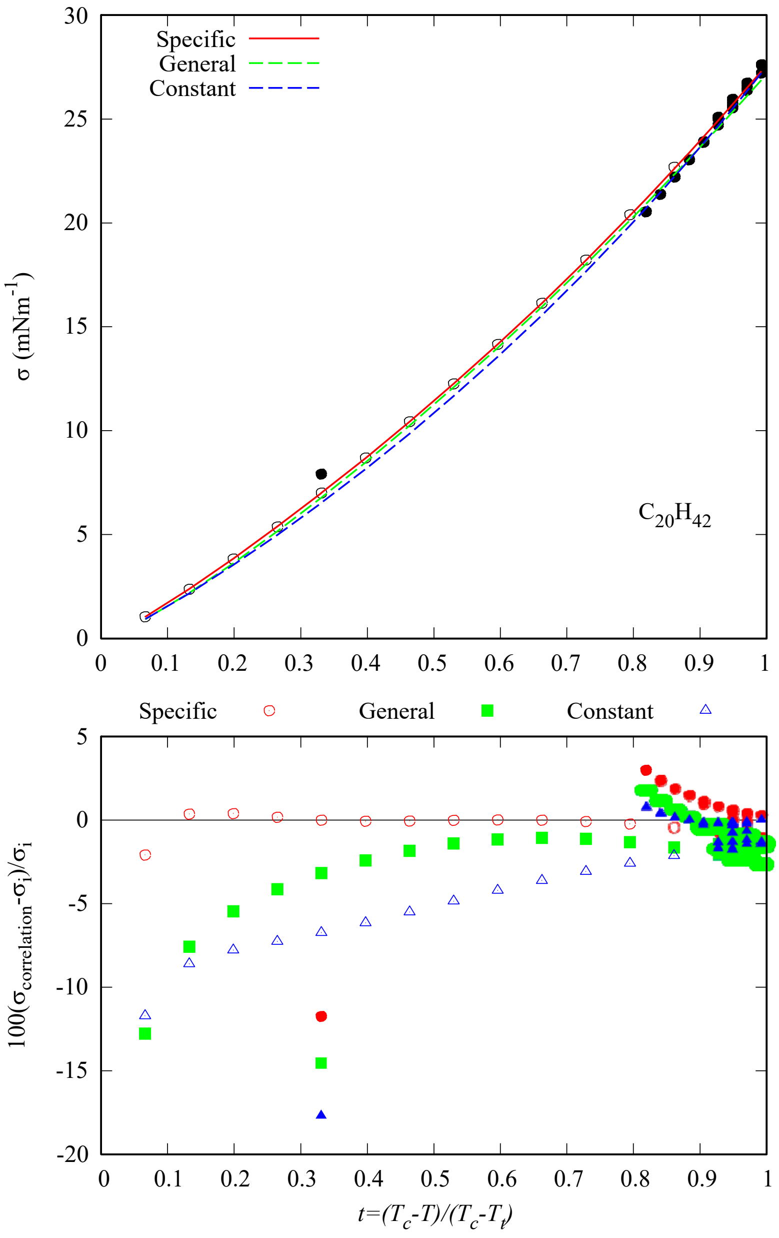

Figure S20. Surface tension data for *n*-eicosane and percentage deviations from the correlations considered here (lines). The open symbols are the data from Sugden's correlation included in the DIPPR database. Closed symbols represent the fitting set data.

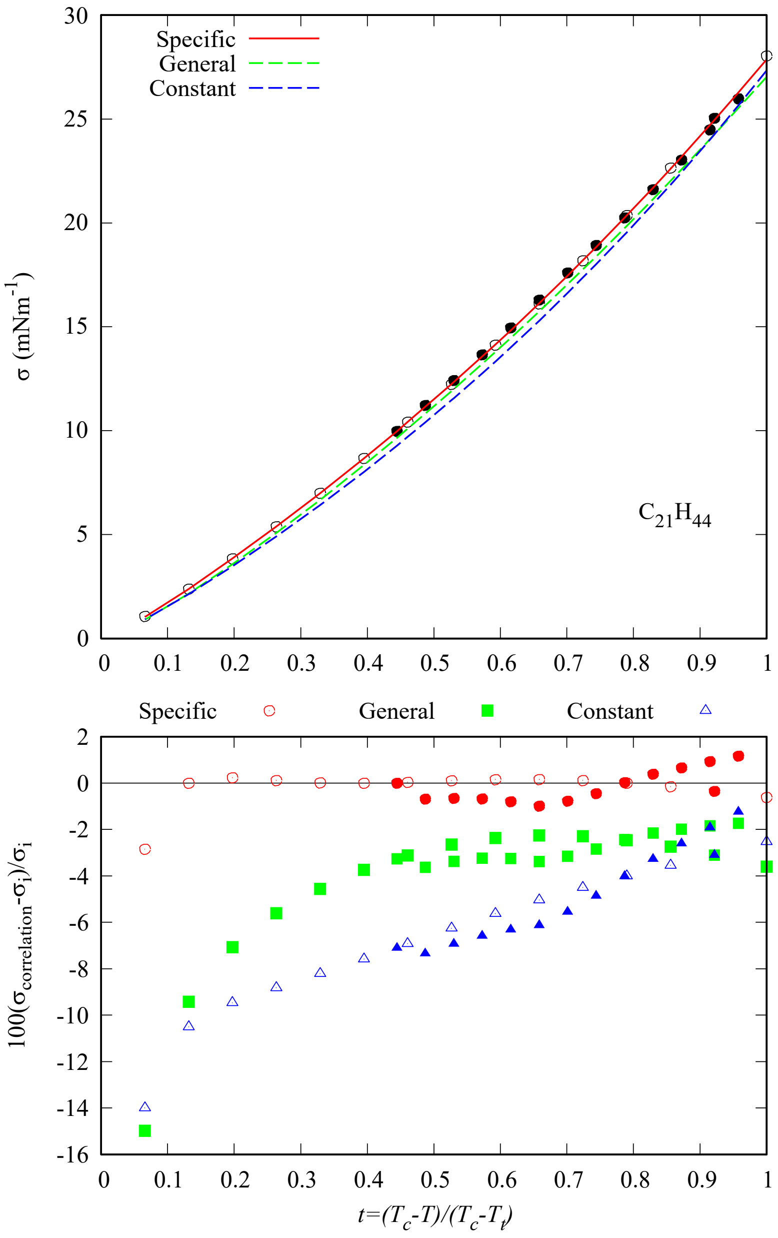

Figure S21. Surface tension data for *n*-heneicosane and percentage deviations from the correlations considered here (lines). The open symbols are the data from Sugden's correlation included in the DIPPR database. Closed symbols represent the fitting set data.

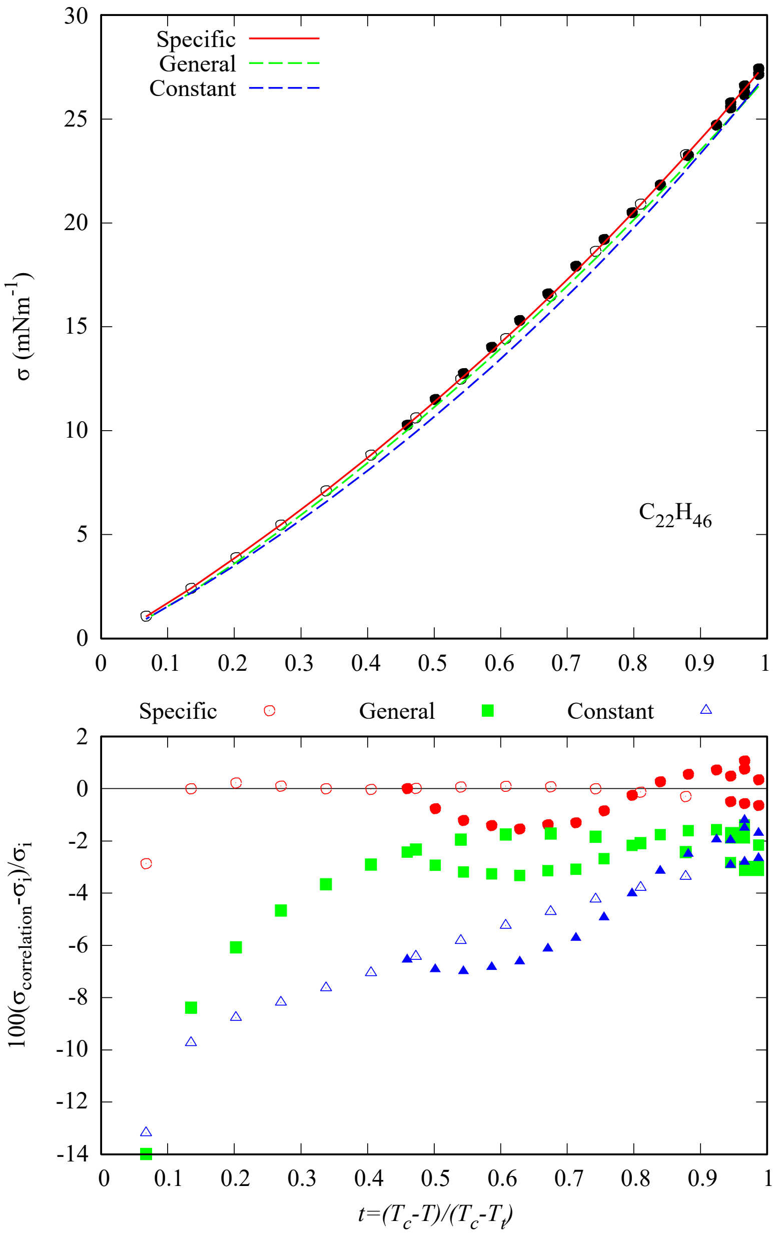

Figure S22. Surface tension data for *n*-docosane and percentage deviations from the correlations considered here (lines). The open symbols are the data from Sugden's correlation included in the DIPPR database. Closed symbols represent the fitting set data.

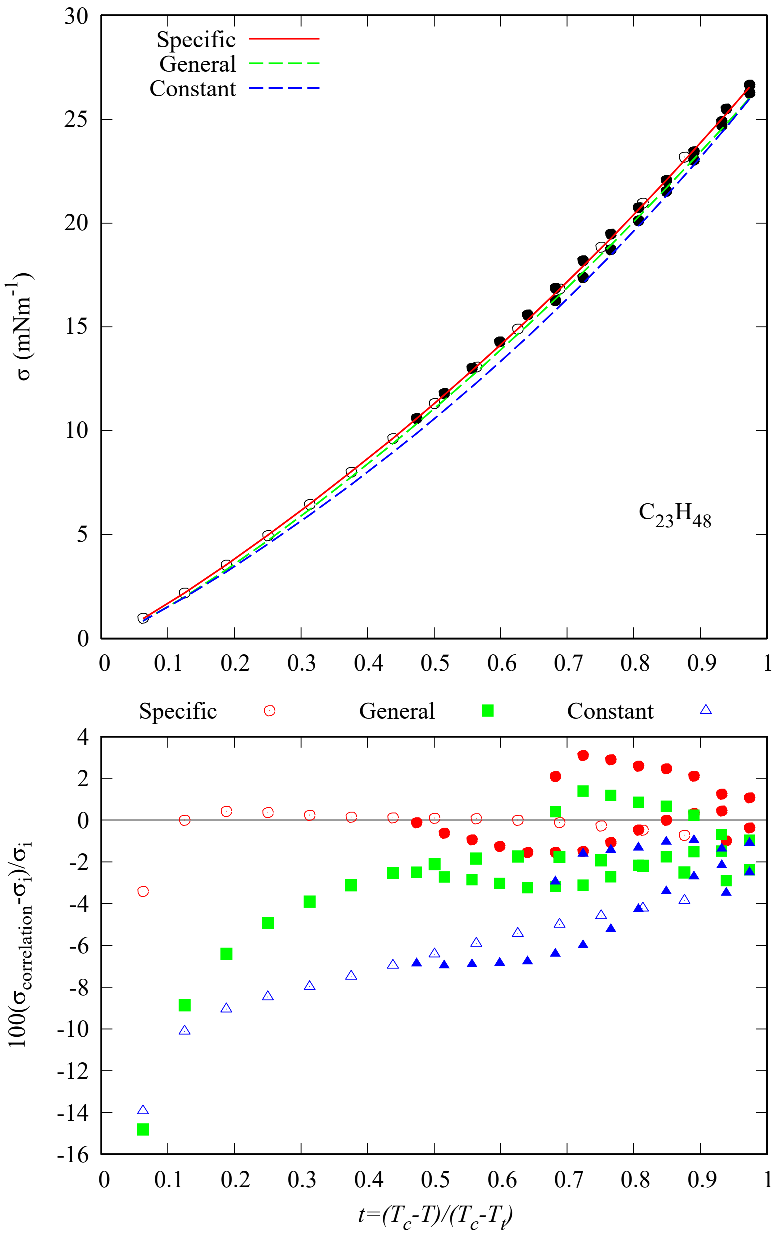

Figure S23. Surface tension data for *n*-tricosane and percentage deviations from the correlations considered here (lines). The open symbols are the data from Sugden's correlation included in the DIPPR database. Closed symbols represent the fitting set data.

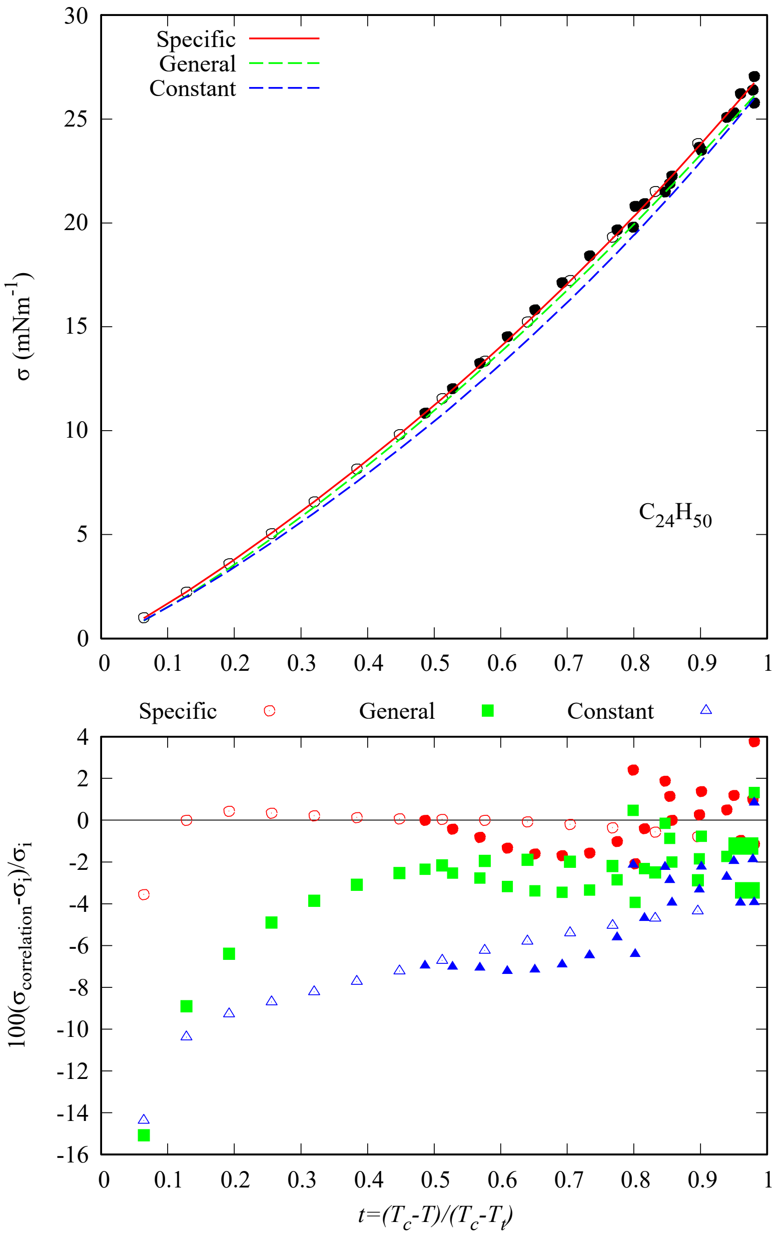

Figure S24. Surface tension data for *n*-tetracosane and percentage deviations from the correlations considered here (lines). The open symbols are the data from Sugden's correlation included in the DIPPR database. Closed symbols represent the fitting set data.

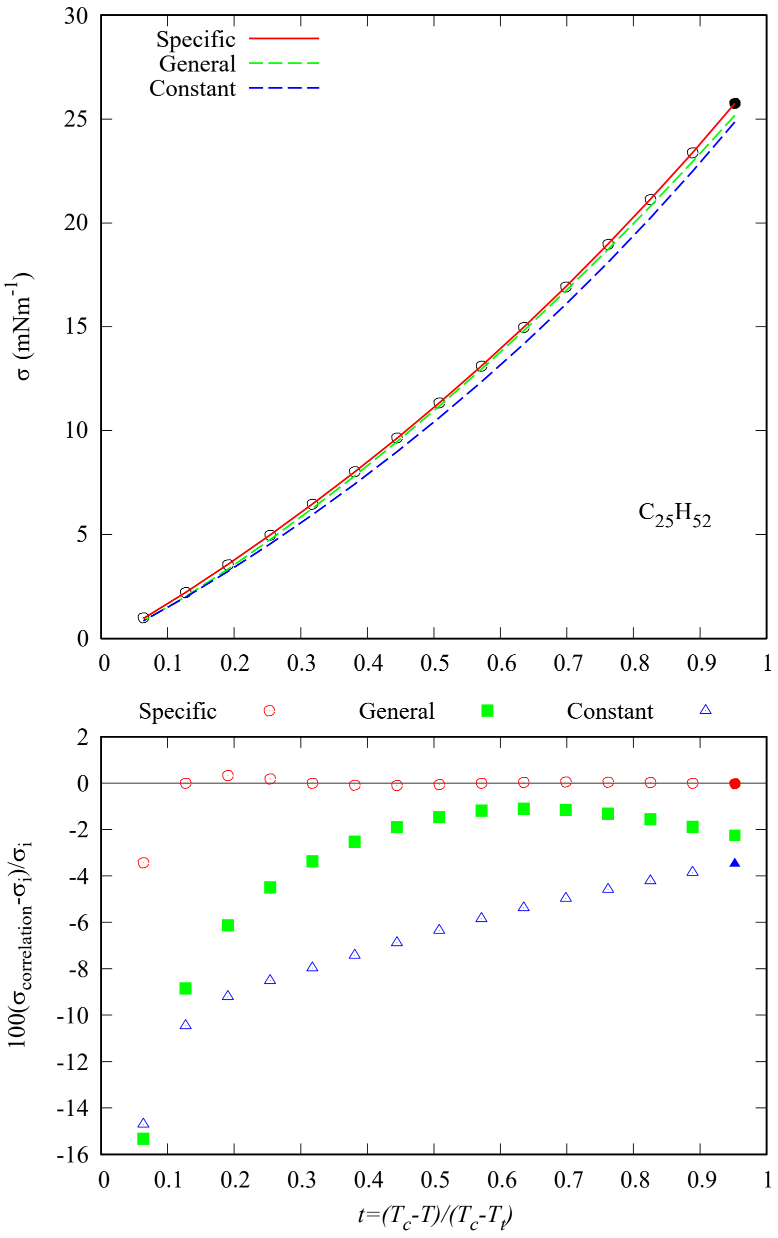

Figure S25. Surface tension data for *n*-pentacosane and percentage deviations from the correlations considered here (lines). The open symbols are the data from Sugden's correlation included in the DIPPR database. Closed symbols represent the fitting set data.

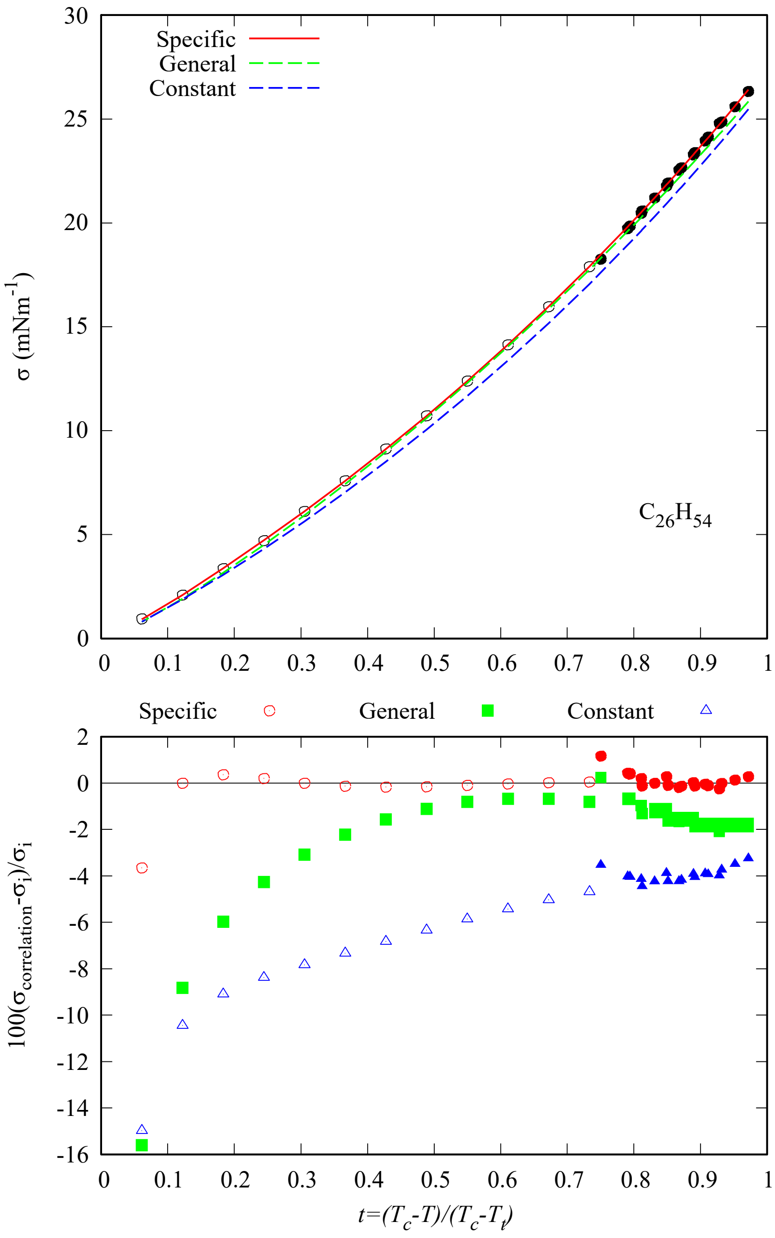

Figure S26. Surface tension data for *n*-hexacosane and percentage deviations from the correlations considered here (lines). The open symbols are the data from Sugden's correlation included in the DIPPR database. Closed symbols represent the fitting set data.

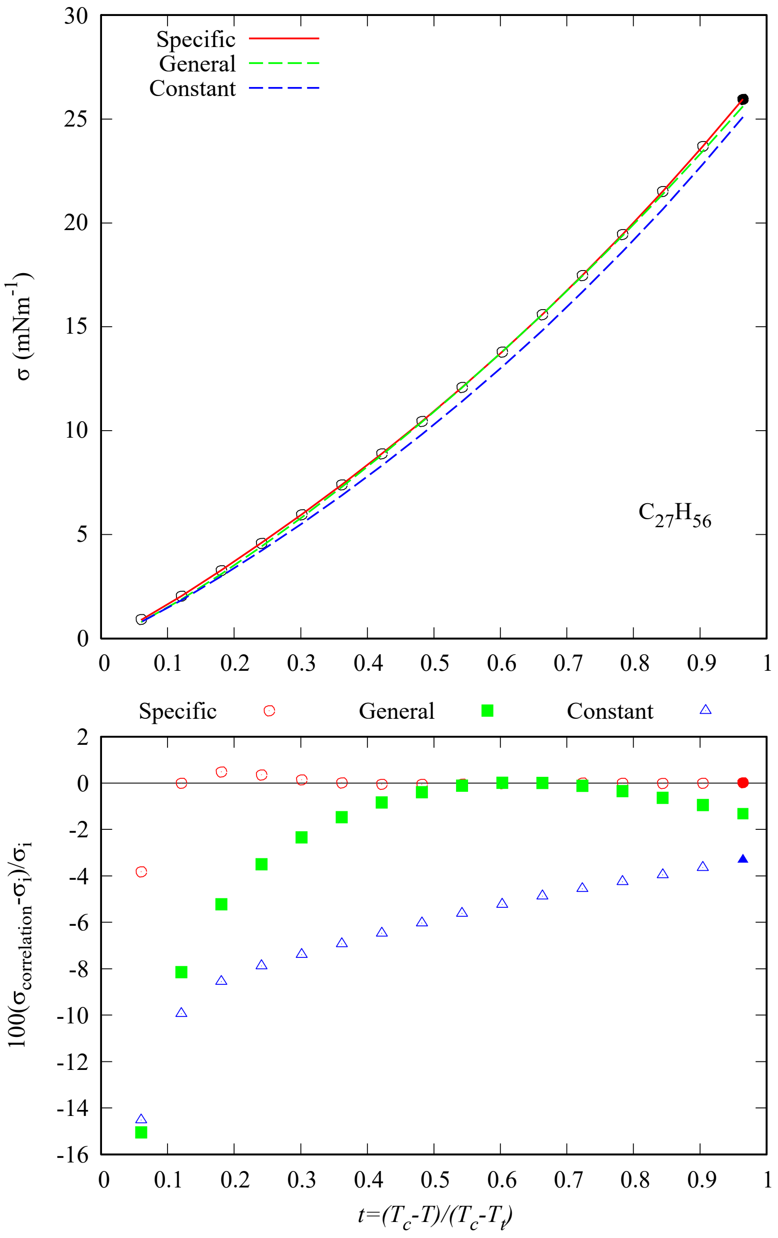

Figure S27. Surface tension data for *n*-heptacosane and percentage deviations from the correlations considered here (lines). The open symbols are the data from Sugden's correlation included in the DIPPR database. Closed symbols represent the fitting set data.

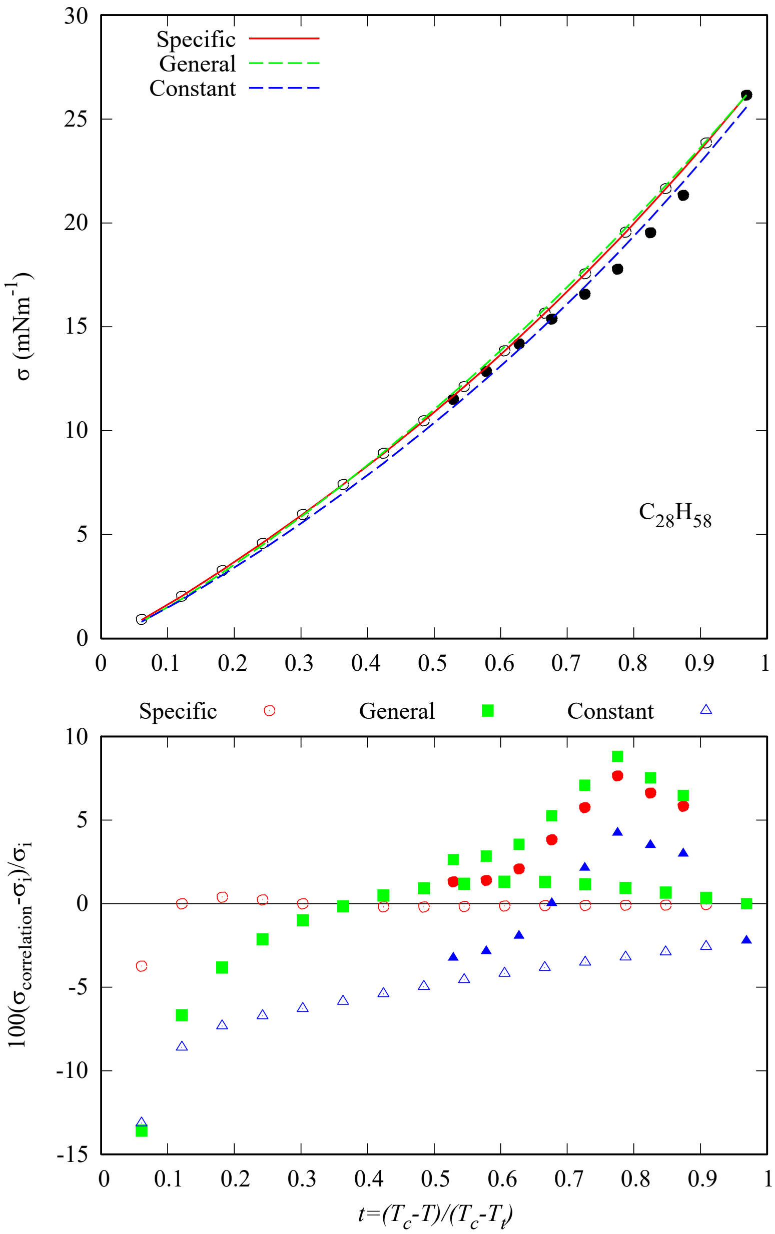

Figure S28. Surface tension data for *n*-octacosane and percentage deviations from the correlations considered here (lines). The open symbols are the data from Sugden's correlation included in the DIPPR database. Closed symbols represent the fitting set data.

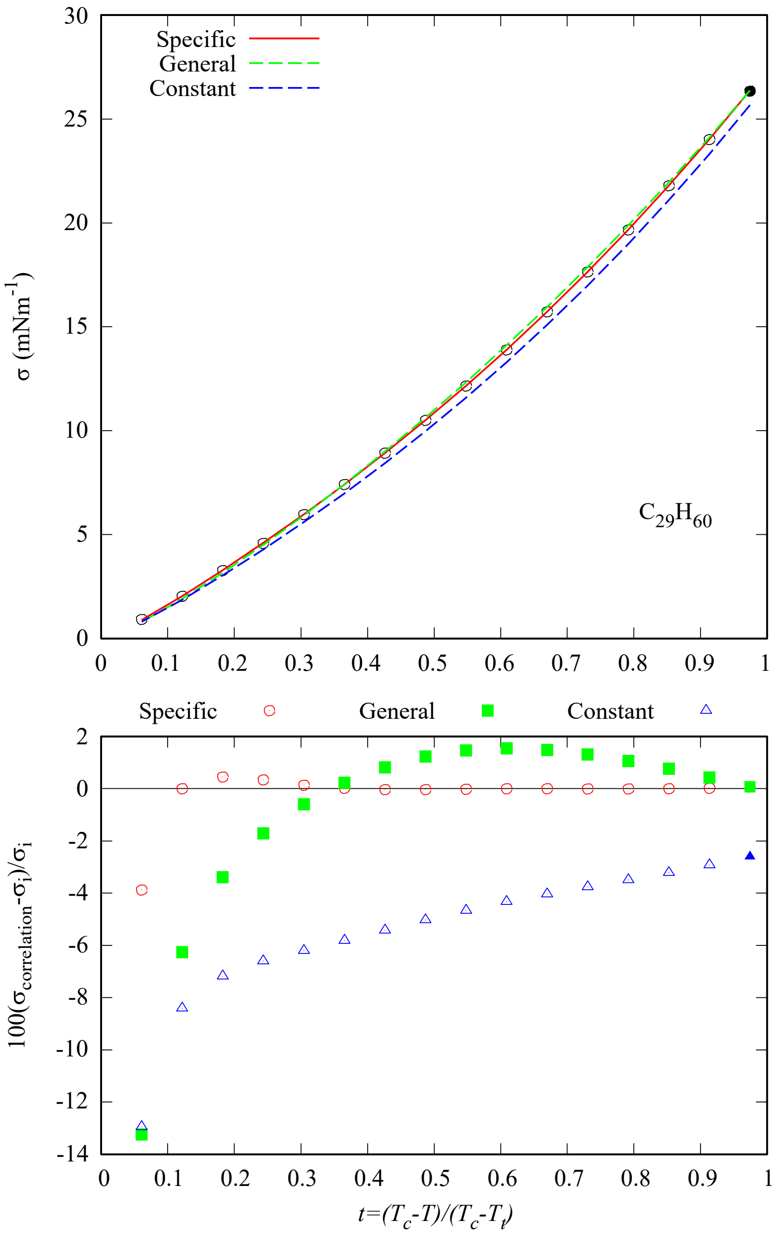

Figure S29. Surface tension data for *n*-nonacosane and percentage deviations from the correlations considered here (lines). The open symbols are the data from Sugden's correlation included in the DIPPR database. Closed symbols represent the fitting set data.

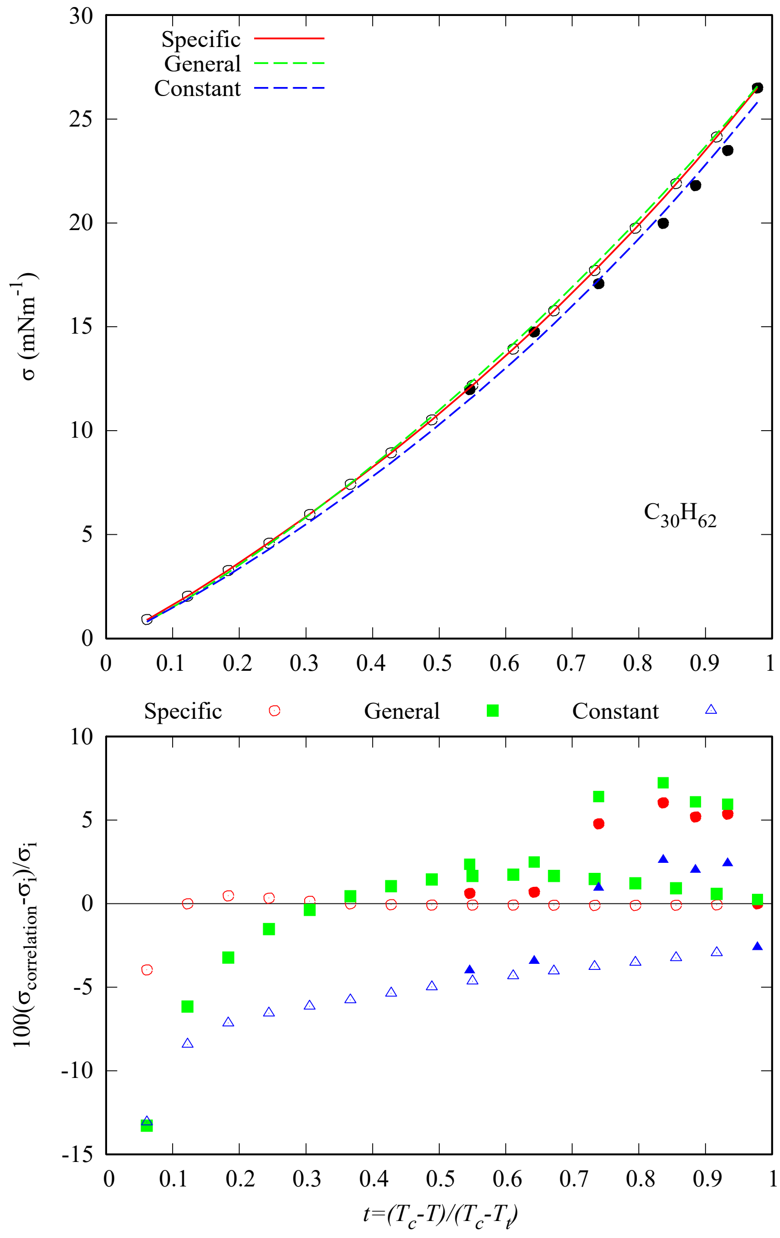

Figure S30. Surface tension data for *n*-triacontane and percentage deviations from the correlations considered here (lines). The open symbols are the data from Sugden's correlation included in the DIPPR database. Closed symbols represent the fitting set data.

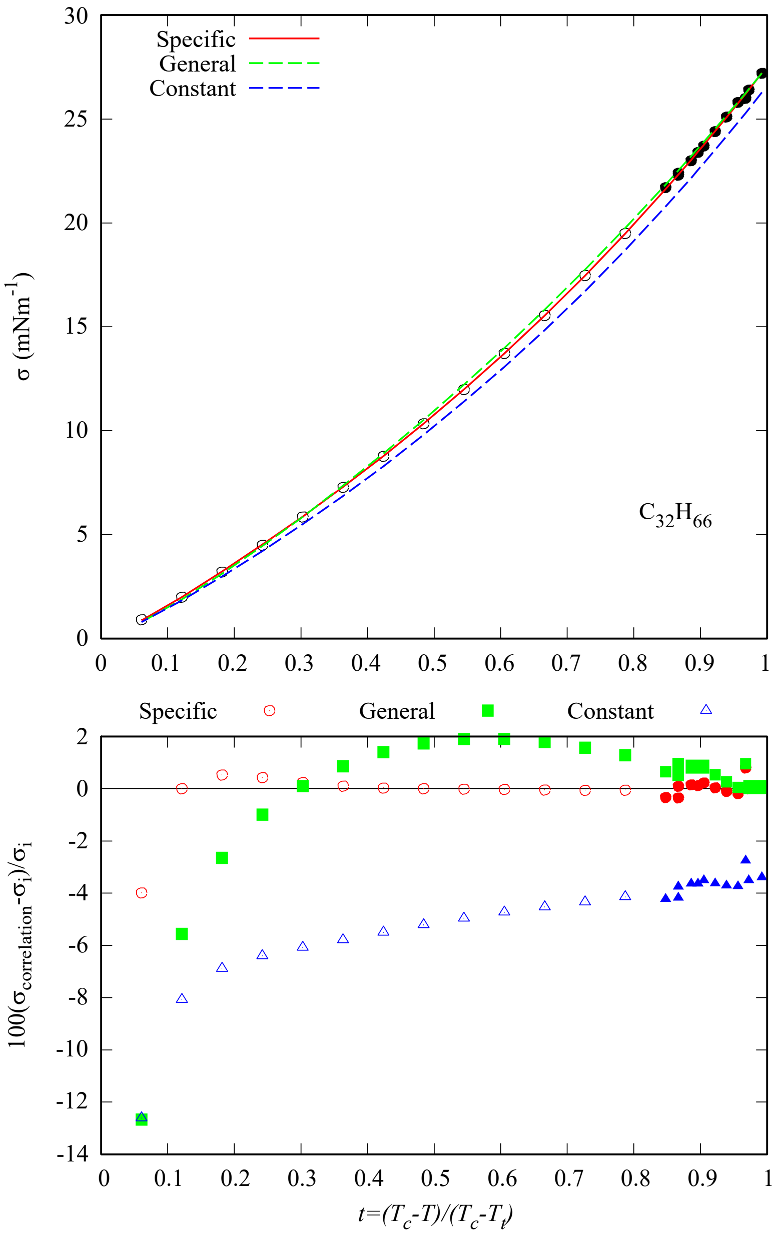

Figure S31. Surface tension data for *n*-dotriacontane and percentage deviations from the correlations considered here (lines). The open symbols are the data from Sugden's correlation included in the DIPPR database. Closed symbols represent the fitting set data.

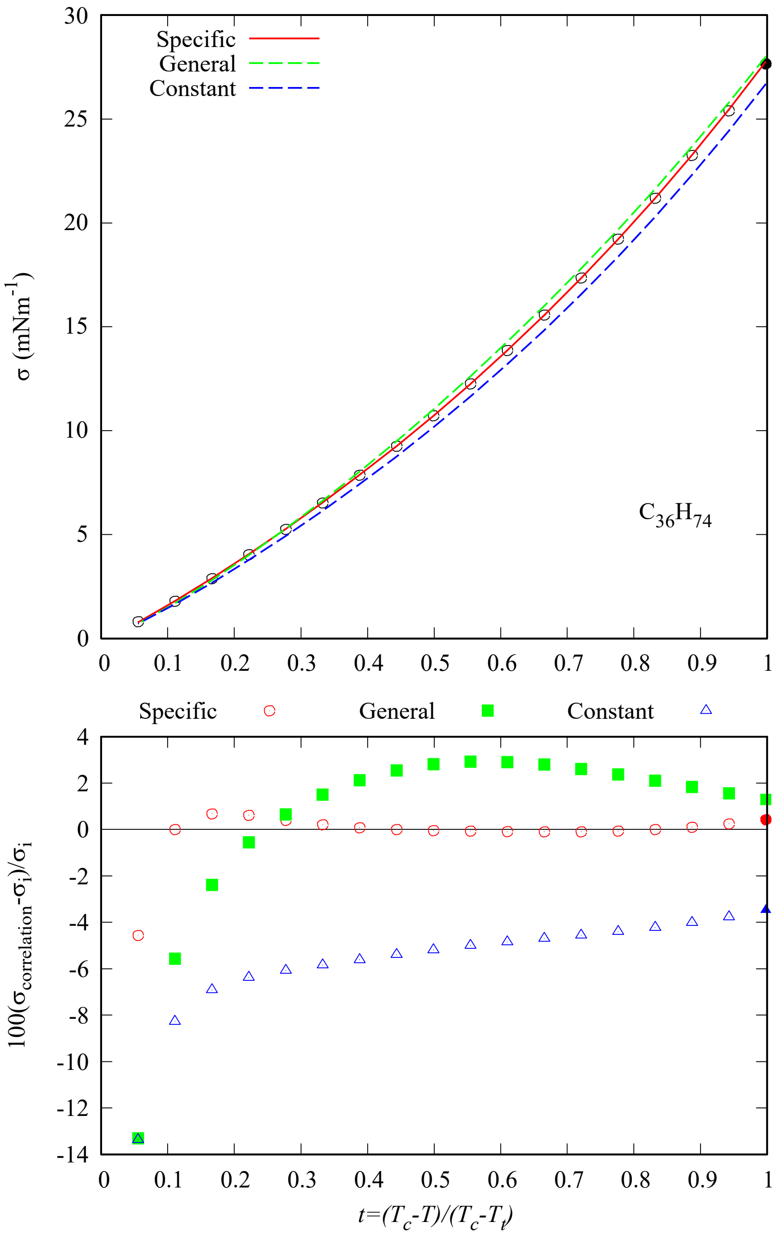

Figure S32. Surface tension data for *n*-hexatriacontane and percentage deviations from the correlations considered here (lines). The open symbols are the data from Sugden's correlation included in the DIPPR database. Closed symbols represent the fitting set data.

Table S1. Statistical figures of the global correlation when m_0 = 4.99955, m_2 = −3.45186 and $m_1 = 0.625431x^{-0.452929} + 3.52179x^{0.405698}$ (all in 10^{-17} mol$^{2/3}$ units), with x being the acentric factor. CN is the carbon number, N the number of data, MAPD the mean absolute percentage deviation, MD the mean deviation, PD_m the maximum absolute percentage deviation, and t_{PDm} is the reduced temperature of the maximum percentage deviation. The subscript fit is added for those figures calculated with the fitting set.

CN	N/N_{fit}	$MAPD/MAPD_{fit}$ (%)	MD/MD_{fit} (%)	$PD_m/PD_{m\ fit}$ (%)	$t_{PDm}/t_{PDm_{fit}}$
1	127/126	2.39/2.37	0.09/ 0.13	14.17/14.17	0.11/ 0.11
2	163/160	6.33/6.17	-5.97/-5.91	20.64/11.87	0.01/ 0.04
3	193/191	2.47/2.28	-1.64/-1.44	26.98/24.35	0.01/ 0.06
4	126/118	6.39/4.17	-0.45/-3.13	64.95/11.78	0.01/ 1.00
5	149/143	3.36/2.22	0.44/-0.82	51.58/11.53	0.01/ 0.02
6	270/269	2.73/2.70	-1.18/-1.23	13.47/13.47	0.07/ 0.07
7	363/357	1.88/1.74	0.66/ 0.50	14.95/14.95	0.05/ 0.05
8	196/194	1.96/1.64	-0.54/-0.89	36.19/6.09	0.01/ 0.19
9	78	0.88	0.27	4.97	0.54
10	149	2.02	1.44	10.54	0.12
11	60	2.44	2.42	14.18	0.30
12	100	3.06	3.04	8.12	0.91
13	48	2.48	2.48	5.95	0.69
14	49	1.47	1.34	5.37	0.72
15	40	2.60	2.60	6.36	0.74
16	127/117	1.97/1.94	0.60/ 0.65	10.45/10.45	0.40/ 0.40
17	44/ 34	1.45/1.13	0.55/ 0.52	6.84/3.64	0.07/ 0.78
18	39/ 29	1.51/1.19	-0.11/ 0.02	8.85/2.99	0.07/ 0.79
19	23/ 12	2.08/1.51	-0.02/ 0.27	10.01/4.65	0.07/ 0.34
20	38/ 25	1.94/1.90	-1.23/-1.05	12.37/12.37	0.33/ 0.33
21	28/ 14	2.15/1.35	-2.15/-1.35	13.44/2.53	0.07/ 0.92
22	32/ 19	1.71/1.33	-1.68/-1.33	12.41/2.90	0.07/ 0.99
23	36/ 22	1.74/1.35	-1.02/-0.22	13.32/2.98	0.06/ 0.72
24	36/ 22	1.82/1.38	-1.60/-1.04	13.61/3.36	0.06/ 0.98
25	15/ 1	2.30/1.94	-1.90/-1.94	13.86/1.94	0.06/ 0.95
26	31/ 19	1.64/0.93	-1.10/-0.65	14.18/1.75	0.06/ 0.97
27	16/ 1	2.30/1.30	-0.97/-1.30	13.67/1.30	0.06/ 0.96
28	24/ 9	4.33/6.43	2.82/ 6.43	12.08/10.26	0.06/ 0.78
29	16/ 1	2.89/0.18	0.74/ 0.18	11.80/0.18	0.06/ 0.97
30	22/ 7	3.97/5.51	2.44/ 5.51	11.81/8.24	0.06/ 0.84
32	25/ 12	2.29/0.94	1.07/ 0.93	11.23/1.63	0.06/ 0.87
36	18/ 1	3.74/1.15	1.97/ 1.15	11.96/1.15	0.06/ 1.00
Overall	2681/3427			64.95/24.35	
	N_{fluid}	OMAPD/ OMAPD $_{fit}$	OMD/ OMD $_{fit}$		
	32	2.57/2.18	0.11/0.24		

Table S2. Statistical figures of the global correlation when $m_0 = 4.91156$, $m_2 = -2.6442$ and $m_1 = 1.09159x^{-0.53364} + 2.68771x^{0.401163}$ (all in 10^{-17} mol$^{2/3}$ units), with x being the critical volume. CN is the carbon number, N the number of data, MAPD the mean absolute percentage deviation, MD the mean deviation, PD_m the maximum absolute percentage deviation, and t_{PDm} is the reduced temperature of the maximum percentage deviation. The subscript fit is added for those figures calculated with the fitting set.

CN	N/N_{fit}	$MAPD/MAPD_{fit}$ (%)	MD/MD_{fit} (%)	$PD_m/PD_{m\ fit}$ (%)	$t_{PDm}/t_{PDm_{fit}}$
1	127/126	5.43/5.40	-5.43/-5.40	18.65/18.65	0.11/ 0.11
2	163/160	3.38/3.16	0.83/ 1.02	20.71/11.38	0.01/ 0.04
3	193/191	2.00/1.81	0.46/ 0.68	27.36/24.37	0.01/ 0.06
4	126/118	4.20/1.92	1.92/-0.51	63.50/9.09	0.01/ 0.03
5	149/143	2.42/1.30	1.96/ 0.83	49.93/10.17	0.01/ 0.02
6	270/269	1.52/1.49	-0.28/-0.32	11.46/11.46	0.07/ 0.07
7	363/357	1.18/1.05	0.80/ 0.67	12.63/12.63	0.05/ 0.05
8	196/194	1.33/1.02	-0.47/-0.80	34.16/3.56	0.01/ 0.77
9	78	0.49	0.34	3.13	0.80
10	149	1.35	0.72	6.97	0.12
11	60	1.42	1.42	10.04	0.30
12	100	1.94	1.91	7.84	0.91
13	48	1.32	1.32	3.42	0.69
14	49	0.81	0.46	4.67	0.40
15	40	1.54	1.54	3.95	0.74
16	127/117	1.73/1.61	-0.51/-0.28	10.01/6.28	0.08/ 0.46
17	44/ 34	1.32/0.85	-1.12/-0.60	9.90/1.68	0.07/ 0.78
18	39/ 29	1.77/1.02	-1.66/-0.89	11.86/2.48	0.07/ 0.99
19	23/ 12	2.56/1.48	-2.30/-0.98	12.93/8.45	0.07/ 0.34
20	38/ 25	2.92/2.03	-2.73/-1.75	15.85/15.85	0.33/ 0.33
21	28/ 14	4.56/3.40	-4.56/-3.40	16.20/4.75	0.07/ 0.49
22	32/ 19	3.71/2.78	-3.71/-2.78	15.20/4.14	0.07/ 0.54
23	36/ 22	3.24/2.06	-3.06/-1.78	15.99/3.91	0.06/ 0.64
24	36/ 22	3.43/2.36	-3.32/-2.18	16.23/4.11	0.06/ 0.80
25	15/ 1	4.35/1.93	-4.35/-1.93	16.45/1.93	0.06/ 0.95
26	31/ 19	2.58/1.24	-2.58/-1.24	16.69/1.76	0.06/ 0.93
27	16/ 1	3.21/0.91	-3.21/-0.91	16.16/0.91	0.06/ 0.96
28	24/ 9	3.49/4.81	0.64/ 4.81	14.65/8.82	0.06/ 0.78
29	16/ 1	2.49/0.68	-1.47/ 0.68	14.32/0.68	0.06/ 0.97
30	22/ 7	3.35/4.66	0.61/ 4.66	14.31/7.60	0.06/ 0.84
32	25/ 12	2.17/1.40	0.06/ 1.40	13.62/2.00	0.06/ 0.97
36	18/ 1	3.32/2.80	0.54/ 2.80	14.15/2.80	0.06/ 1.00
Overall	2681/2427			63.50/24.37	
	N_{fluid}	OMAPD/ OMAPD $_{fit}$	OMD/ OMD $_{fit}$		
	32	2.52/1.94	-0.79/-0.02		

Table S3. Statistical figures of the global correlation when $m_0 = 5.37325$, $m_2 = 2.05972$ and $m_1 = 0.662073x^{-0.595946} + 4.84453x^{0.542305}$ (all in 10^{-17} mol$^{2/3}$ units), with x being the liquid molar volume at 298.15 K and 101325 Pa. CN is the carbon number, N the number of data, MAPD the mean absolute percentage deviation, MD the mean deviation, PD_m the maximum absolute percentage deviation, and t_{PDm} is the reduced temperature of the maximum percentage deviation. The subscript fit is added for those figures calculated with the fitting set.

CN	N/N_{fit}	MAPD/MAPD$_{fit}$ (%)	MD/MD$_{fit}$ (%)	PD$_m$ /PD$_{m\ fit}$ (%)	$t_{PDm}/t_{PDm_{fit}}$
1	127/126	3.35/3.33	-1.43/-1.40	16.82/16.82	0.11/ 0.11
2	163/160	3.44/3.22	-2.54/-2.44	19.07/11.63	0.01/ 0.15
3	193/191	2.08/1.91	-0.47/-0.28	25.46/23.76	0.01/ 0.06
4	126/118	4.79/2.29	1.76/-0.94	68.36/11.25	0.01/ 0.03
5	149/143	2.97/1.72	1.77/ 0.47	54.88/12.73	0.01/ 0.02
6	270/269	1.97/1.93	-0.54/-0.59	12.54/12.32	0.01/ 0.04
7	363/357	1.43/1.26	0.59/ 0.41	14.97/14.01	0.01/ 0.05
8	196/194	1.51/1.16	-0.52/-0.89	38.48/3.59	0.01/ 0.21
9	78	0.65	0.46	3.40	0.80
10	149	1.29	0.69	6.70	0.12
11	60	1.35	1.33	8.28	0.30
12	100	2.01	1.94	8.54	0.91
13	48	1.41	1.41	2.47	0.69
14	49	0.99	0.66	6.51	0.40
15	40	1.77	1.73	3.08	0.74
16	127/117	1.53/1.28	-0.50/-0.17	9.79/4.87	0.08/ 0.81
17	44/ 34	1.65/0.86	-1.58/-0.77	9.60/2.53	0.07/ 0.60
18	39/ 29	2.05/0.93	-2.03/-0.89	11.60/1.80	0.07/ 0.99
19	23/ 12	3.20/1.41	-3.10/-1.22	12.57/10.33	0.07/ 0.34
20	38/ 25	3.20/1.80	-3.12/-1.68	17.58/17.58	0.33/ 0.33
21	28/ 14	5.58/4.35	-5.58/-4.35	15.84/6.64	0.07/ 0.49
22	32/ 19	4.55/3.34	-4.55/-3.34	14.88/5.91	0.07/ 0.54
23	36/ 22	4.00/2.54	-3.99/-2.51	15.58/5.53	0.06/ 0.52
24	36/ 22	4.25/2.94	-4.12/-2.74	15.85/5.39	0.06/ 0.61
25	15/ 1	5.41/1.59	-5.41/-1.59	16.05/1.59	0.06/ 0.95
26	31/ 19	3.21/1.44	-3.21/-1.44	16.23/1.87	0.06/ 0.81
27	16/ 1	4.23/0.48	-4.23/-0.48	15.68/0.48	0.06/ 0.96
28	24/ 9	3.48/3.92	-0.39/ 3.92	14.16/8.05	0.06/ 0.78
29	16/ 1	2.90/1.20	-2.47/ 1.20	13.85/1.20	0.06/ 0.97
30	22/ 7	3.32/4.17	-0.28/ 4.17	13.83/7.25	0.06/ 0.84
32	25/ 12	2.24/1.59	-0.48/ 1.59	13.13/2.50	0.06/ 0.97
36	18/ 1	3.01/3.68	-0.23/ 3.68	13.50/3.68	0.06/ 1.00
Overall	2681/2427			68.36/23.76	
	N_{fluid}	OMAPD/ OMAPD $_{fit}$	OMD/ OMD $_{fit}$		
	32	2.78/1.99	-1.20/-0.13		
